# Oligomeric Forms of Human Amyloid-Beta(1–42) Inhibit Antigen Presentation

**DOI:** 10.3389/fimmu.2020.01029

**Published:** 2020-06-05

**Authors:** Christoph Gericke, Anna Mallone, Britta Engelhardt, Roger M. Nitsch, Maria Teresa Ferretti

**Affiliations:** ^1^Institute for Regenerative Medicine - IREM, University of Zurich, Schlieren, Switzerland; ^2^Zurich Neuroscience Center (ZNZ), Zurich, Switzerland; ^3^Theodor Kocher Institute, University of Bern, Bern, Switzerland; ^4^Neurimmune AG, Schlieren, Switzerland

**Keywords:** Alzheimer's Disease, antigen presentation, amyloid-beta (Aβ) 1-42, microglia, CNS border-associated macrophages, CNS dendritic cells, T-cell activation and proliferation

## Abstract

Genetic, clinical, biochemical and histochemical data indicate a crucial involvement of inflammation in Alzheimer's disease (AD), but harnessing the immune system to cure or prevent AD has so far proven difficult. Clarifying the cellular heterogeneity and signaling pathways associated with the presence of the AD hallmarks beta-amyloid and tau in the brain, would help to identify potential targets for therapy. While much attention has been so far devoted to microglia and their homeostatic phagocytic activity, additional cell types and immune functions might be affected in AD. Beyond microglia localized in the brain parenchyma, additional antigen-presenting cell (APC) types might be affected by beta-amyloid toxicity. Here, we investigated potential immunomodulatory properties of oligomeric species of beta-amyloid-peptide (Aβ) on microglia and putative APCs. We performed a comprehensive characterization of time- and pathology-dependent APC and T-cell alterations in a model of AD-like brain beta-amyloidosis, the APP-PS1-dE9 mouse model. We show that the deposition of first beta-amyloid plaques is accompanied by a significant reduction in MHC class II surface levels on brain APCs. Furthermore, taking advantage of customized *in vitro* systems and RNAseq, we demonstrate that a preparation containing various forms of oligomeric Aβ1-42 inhibits antigen presentation by altering the transcription of key immune mediators in dendritic cells. These results suggest that, beyond their neurotoxic effects, certain oligomeric Aβ forms can act as immunomodulatory agents on cerebral APCs and interfere with brain antigen presentation. Impaired brain immune surveillance might be one of the factors that facilitate Aβ and tau spreading in AD.

## Introduction

Alzheimer's disease (AD) is a neurodegenerative disease leading to progressive loss of cognitive and memory functions, ultimately resulting in dementia syndrome. AD brains present with the accumulation of beta-amyloid plaques composed of aggregated neurotoxic beta-amyloid peptide (Aβ) and neurofibrillary tangles of abnormally phosphorylated tau protein (1). Genetic evidence has unequivocally identified immune pathways as key drivers of AD susceptibility (2). In fact, several neuroinflammatory changes in the phenotype and functionality of immune cells occur in AD (3). Prominent examples are microglia, the innate resident immune cells of the brain parenchyma. Microglia migrate toward beta-amyloid plaques (4), surround them and initiate inflammasome activation (5), secretion of pro-inflammatory cytokines (6, 7) as well as upregulation of phagocytosis and antigen presentation-related genes (8). Being a crucial regulator of brain homeostasis, the immune system could be an ideal target for treatment. However, past attempts to inhibit (9, 10) or harness (11) immune pathways proved to be non-beneficial in AD patients. Such clinical failures underscore our yet incomplete understanding of the cellular heterogeneity and signaling pathways involved in AD. In particular, recent observations of a dural lymphatic system (12, 13) as well as lymphatic vessels being the major outflow pathway for cerebrospinal fluid (CSF) (14), indicate that the brain might be effectively connected with secondary lymphoid organs and subjected to immune surveillance, with a variety of CNS and blood-derived immune cells involved (15).

Brain immune surveillance relies on antigen-specific memory T-cells, which monitor the CNS by circulating through CSF-filled compartments, searching for their cognate antigenic material presented on antigen-presenting cells (APCs) via major histocompatibility complexes (MHC class I or II). Professional APCs with high surface MHC class II (MHC-II) expression are normally absent from brain parenchyma, but present in CSF-filled compartments, including leptomeningeal and choroid plexus dendritic cells (DCs) (16) as well as non-parenchymal macrophages at CNS boundaries (perivascular spaces, leptomeninges and choroid plexus) (17). However, parenchymal APCs such as microglia can up-regulate MHC-II surface levels after activation (18). Reactivation of T-cells upon detection of cognate antigens presented by APCs licenses them for parenchyma access (19) and leads to downstream effector functions. T cell effects vary according to the CNS milieu, ranging from homeostatic regulation of neurogenesis, removal of pathogens and cancer cells, to synaptic pruning and neuronal toxicity (20–23). Alterations of immune surveillance are closely linked to brain pathology (24) but their role in AD is not fully elucidated (25). In the context of AD, impaired immune surveillance might lead to immune evasion of Aβ aggregates and uncontrolled plaque deposition. Indeed, it has been shown that mice lacking MHC-II harbor increased beta-amyloid plaques and inflammation (26). On the other hand, overt immune activation of patrolling T-cells might cause neuronal toxicity.

In a previous study, we have shown that late-stage beta-amyloid accumulation in aged transgenic mouse models of AD-like cerebral beta-amyloidosis is accompanied by reduced MHC-II expression per single APC and by reduced frequency of T-cells secreting pro-inflammatory cytokines IFNγ and TNFα (27). These results suggested that beta-amyloid aggregation is associated with impaired brain immune surveillance, potentially via inhibitory effects on APCs.

Here, we explored the immunomodulatory effects of Aβ *ex vivo* and *in vitro*. First, we defined the precise temporal relationship between beta-amyloid accumulation and MHC-II expression as well as T-cell alterations by using transgenic mouse models with robust beta-amyloid deposition. Second, to prove a direct effect of beta-amyloid on APCs, we used *in vitro* assays of antigen presentation with primary DCs to test the immune-altering properties of human recombinant oligomeric Aβ species.

## Materials and Methods

### Transgenic Animals

All mice were on a congenic C57BL/6J background. We used amyloid precursor protein (APP)-overexpressing heterozygous Swedish/APP-PS1-dE9 mice (B6.Cg-Tg (APPswe, PSEN1dE9)85Dbo/J, termed “APP-PS1” throughout this manuscript), co-expressing KM670/671NL-mutated chimeric mouse/human APP (the so-called Swedish mutation) and exon9-deleted presenilin-1 (PS1-dE9) under the control of the mouse prion protein promoter (28). Within each experimental group, equal numbers of genotypes (APP-PS1 transgenic vs. age-matched, non-transgenic littermate controls) and genders were distributed. The covariate 'gender' had no effect on age- and genotype-analysis. Heterozygous T-cell receptor transgenic B6.Cg-Tg(TcraTcrb)425Cbn/J mice (termed “OT-II” throughout this manuscript) expressing a T-cell receptor specific for chicken ovalbumin (OVA) in the context of MHC-II (29) were used as T-cell source for antigen presentation assays. Non-transgenic C57BL/6J mice were used as source for bone marrow-derived progenitor cells for antigen presentation assays. The mice were kept under OHB-conditions on a 12 h light, 12 h dark cycle. Food and water were provided *ad libitum*. All animal experiments were approved by the Swiss cantonal veterinary office (Canton Zurich, license numbers 145/2014 and 064/2017). This manuscript adheres to the ARRIVE guidelines.

### Mouse Tissue Extraction

Mice were deeply anesthetized via ketamine-xylazine (ketamine: 20 mg/ml; xylazine: 2 mg/ml) injection at 10 μl per gram bodyweight, followed by transcardial perfusion for 2 min with cold phosphate buffered saline (PBS). After complete drainage of blood circulation, brains were immediately extracted and brain regions of interest were dissected. For these experiments, we used only the cerebrum, i.e., the brain without cerebellum and olfactory bulbs. Control tissues such as spleen and inguinal lymph nodes were extracted and analyzed in order to exclude systemic effects of APP overexpression in APP-PS1 animals. Animal organs were only included if no apparently pathological symptoms were observed during the organ extraction (such as abnormally increased spleen as sign of an ongoing infection, or tumors).

### Histology and Thioflavin-S Staining

Cerebrum hemispheres were fixed in 4% (w/v) paraformaldehyde (PFA) in 0.1 M phosphate buffer for 24 h at 4°C and subsequently transferred into a 30% (w/v) sucrose solution in 0.1 M phosphate buffer for 72 h at 4°C (cryoprotection). Hemispheres were cut at −20°C into 40 μm-thick coronal slices with a sliding microtome (Microm HM 450, Thermo Scientific). Slices were cryo-protected in 37.5% (w/v) sucrose and 37.5% (v/v) ethylene glycol in 0.1 M phosphate buffer and stored at −20°C.

Free-floating slices were rinsed with distilled water for 2 min, followed by a treatment in 1% (w/v) Thioflavin-S (T1892, Sigma) solution in 50% (v/v) ethanol for 5 min at room temperature. After washing steps in 50% (v/v) ethanol (2 × 2 min), distilled water (1 × 2 min) and PBS (1 × 5 min), slices were mounted on microscope slides.

Via fluorescence microscopy (microscope: DM4000B, Leica) and ImageJ imaging analysis software (version 1.52d, with Fiji plugin package, National Institutes of Health, USA) we quantified Thioflavin-S-positive beta-amyloid plaques in an area of 470 × 700 μm (0.33 mm^2^) in the cortex or in the CA1 region of anterior hippocampus.

### Brain Tissue Homogenization

Prefrontal cortices from one hemisphere were dissected, immediately snap frozen in liquid nitrogen and stored at −80°C. In a first step, frozen prefrontal cortices were weighed and homogenized in a 10-fold wet weight amount of TBS (Tris-buffered saline: 150 mM NaCl, 100 mM Tris, pH 8.0 + Complete Protease Inhibitor Cocktail, Roche Diagnostics) using a 1 ml Dounce glass homogenizer with tight-fitting pestle. Homogenates were centrifuged at 100,000 × g for 1 h at 4°C. TBS-soluble supernatants were collected and stored at −80°C (termed “TBS-soluble fraction” throughout this manuscript). The pellets were re-homogenized in TBS containing 2% (w/v) sodium dodecyl sulfate (SDS) and centrifuged at 100,000 × g for 1 h at 8°C. SDS-soluble supernatants were collected and stored at −80°C (termed “SDS-soluble fraction” throughout this manuscript). The pellets were resuspended in 70% (v/v) formic acid, twice sonicated on ice for 30 s and centrifuged at 100,000 × g for 30 min at 4°C. Formic acid-soluble supernatants were collected, lyophilized and reconstituted in sample diluent of Aβ detection assay (termed “Formic acid-soluble fraction” throughout this manuscript).

### Meso Scale Discovery (MSD) Analysis

Levels of Aβ1-38, Aβ1-40, and Aβ1-42 were measured in all homogenate fractions of hemispheric prefrontal cortices using electrochemiluminescence assays (96-well MultiSpot Human 6E10 Aβ Triplex Assay, MSD) according to the manufacturer's instructions. Samples were diluted to fit an internal Aβ Triplex standard curve. Plates were analyzed on a MSD SECTOR Imager 600 plate reader and MSD DISCOVERY WORKBENCH software (Version 3.0.17, MESO SCALE DIAGNOSTICS, LLC) with Data Analysis Toolbox was used to calculate sample concentrations by comparing them against the internal standard curve.

### Single Cell Suspensions for Flow Cytometric Analysis

#### Generation of Brain Mononuclear Single Cell Suspensions

Brain hemispheres were minced into small pieces with scalpels and digested with 2 mg/ml Collagenase D (Roche) and 50 μg/ml DNase I (Roche) in HBSS (with Mg^2+^ and Ca^2+^, Gibco, Thermo Scientific) for 30 min at 37°C on a magnetic stirrer. Digestion was stopped on ice and single cell suspensions were generated by mashing and filtering through 100 μm nylon meshes (BD Biosciences). After one centrifugation at 350 × g for 10 min at 4°C, pellets were resuspended in 35% (v/v) Percoll (GE Healthcare Life Sciences, max. density: 1.135 g/ml) in HBSS (without Mg^2+^ and Ca^2+^, Gibco, Thermo Scientific) and centrifuged at 29,000 × g for 30 min at 4°C (LYNX 4000 centrifuge, Sorvall, Thermo Scientific) with SS-34 rotor (Thermo Scientific). Separated myelin layer was aspirated and aqueous mononuclear cell phase was collected. Single cell suspensions were refiltered through 70 μm nylon meshes (BD Biosciences) and washed in HBSS (without Mg^2+^ and Ca^2+^).

#### Generation of Spleen Mononuclear Single Cell Suspensions

Spleens were minced in RPMI-1640 (Gibco, Thermo Scientific) + 2% (v/v) FBS (Gibco, Thermo Scientific, heat-inactivated) and digested with 50 μg/ml DNase I (Roche) at room temperature for 30 min. Digestion was stopped on ice and single cell suspensions were generated by mashing and filtering through 100 μm nylon meshes (BD Biosciences). After one centrifugation at 350 × g for 10 min at 4°C, pellets were resuspended in ammonium-chloride-potassium lysis buffer (ACK buffer: 150 mM NH_4_Cl, 10 mM KHCO_3_, 0.1 mM Na_2_EDTA, pH 7.4, all chemicals from Sigma) and incubated for 4 min on ice in order to lyse splenic erythrocytes. Single cell suspensions were washed in HBSS (without Mg^2+^ and Ca^2+^) + 2% (v/v) FBS (heat-inactivated), refiltered through 70 μm nylon meshes (BD Biosciences) and washed again in HBSS (without Mg^2+^ and Ca^2+^) + 2% (v/v) FBS (heat-inactivated).

#### Generation of Mononuclear Single Cell Suspensions From Lymph Nodes

Inguinal lymph nodes were mashed and filtered through 70 μm nylon meshes (BD Biosciences). Single cell suspensions were washed in HBSS (without Mg^2+^ and Ca^2+^) + 2% (v/v) FBS (heat-inactivated) by centrifugation at 350 × g for 10 min at °C.

### Flow Cytometry

#### Myeloid Cell Panel

Single cell suspensions deriving from brain and spleen tissue were incubated for 30 min at 4°C in Zombie Aqua live/dead exclusion dye (1:1000 from stock in HBSS (without Mg^2+^ and Ca^2+^), LIVE/DEAD™ Fixable Aqua Dead Cell Stain Kit, Molecular Probes, Thermo Scientific). Next, cells were washed in FACS buffer (2% (v/v) FBS (heat-inactivated), 5 mM EDTA, 0.01% (v/v) NaN_3_) and resuspended in Fc receptor blocking antibodies (1:20 from stock in FACS buffer, anti-mouse CD16/32 TruStain fcX, Biolegend). After 5 min incubation, cells were treated with antibody master mix for surface marker staining and incubated for 15 min at 4°C, followed by a last wash in FACS buffer. For myeloid cells (microglia, dendritic cells, macrophages) we used the following fluorophore-conjugated antibodies against surface markers: PE-Cy5.5 anti-CD45 (clone 30-F11, eBioscience, Thermo Scientific), PE-Cy7 anti-CD11b (clone M1/70, eBioscience, Thermo Scientific), APC anti-CD11c (clone N418, BioLegend), Alexa Fluor 488 anti-MHC class II (clone M5/114.15.2, Biolegend).

#### T-Cell Panel

In order to amplify intracellular cytokine production, single cell suspensions generated from brain and lymph nodes were restimulated with 50 ng/ml PMA (Sigma), 1 μg/ml Ionomycin (Sigma) and Brefeldin A (1:1000 from stock, GolgiPlug, BD Biosciences) in RPMI-1640 containing 10% (v/v) FBS (heat-inactivated) for 4 h at 37°C. After restimulation, cells were washed, stained with live/dead exclusion dye, blocked and surface antibody-labeled as described above. For T-cells we used the following fluorophore-conjugated antibodies against surface markers: PE-Cy5.5 anti-CD45 (clone 30-F11, eBioscience, Thermo Scientific), PE-Cy7 anti-CD11b (clone M1/70, eBioscience, Thermo Scientific), APC anti-TCRβ (clone H57-597, BioLegend), APC-Cy7 anti-CD4 (clone GK1.5, BioLegend), Alexa Fluor 700 anti-CD8α (clone 53-6.7, eBioscience, Thermo Scientific). For intracellular cytokine staining, cells were fixed and permeabilized according to manufacturer's instructions (Fixation/Permeabilization and Permeabilization Buffer, eBioscience, Thermo Scientific). Cells were incubated with antibody master mix for cytokine staining in 1x Permeabilization buffer for 30 min at 4°C. We used the following fluorophore-conjugated antibodies against intracellular markers: eFluor 450 anti-IFNγ (clone XMG1.2, eBioscience, Thermo Scientific), FITC anti-TNFα (clone MP6-XT22, eBioscience, Thermo Scientific), PE anti-FoxP3 (clone FJK-16s, eBioscience, Thermo Scientific), APC anti-IL-10 (clone JES5-16E3, eBioscience, Thermo Scientific).

For brain-derived samples we used counting beads in order to determine absolute cell numbers (AccuCheck Counting Beads, Thermo Scientific). Fluorescence-minus-one (FMO) samples were included as negative controls. Cells were acquired on a 16-channel LSR II Fortessa flow cytometer (BD Biosciences). FlowJo (Version 10.0.8, FLOWJO LLC) software was used for flow cytometry data analysis.

### *In vitro* Antigen Presentation Assays

#### Extraction and Cultivation of Bone Marrow Progenitor Cells

Bone marrow-derived progenitor cells were isolated from femurs, tibiae and hip bones of non-transgenic C57BL/6J mice according to established protocols (30). Progenitor cells were cultivated in RPMI-1640 with 10% (v/v) FBS (heat-inactivated), GlutaMAX supplement (1:100 from stock, Gibco, Thermo Scientific), 50 μM β-mercaptoethanol (Sigma) and 200 U/ml murine Granulocyte-Macrophage Colony-Stimulating Factor (GM-CSF, PeproTech) at 0.2 Mio. cells/ml. After 9 days of incubation at 37°C and 5% CO_2_, progenitor cells completely differentiated into immature bone marrow-derived dendritic cells (BM-DCs).

#### BM-DC Maturation and Antigen Presentation

Antigen presentation assays were carried out, with minor adaptations, as previously described (31). Maturation of BM-DCs was achieved by an 18 h LPS-treatment at 100 ng/ml followed by an up-regulation of antigen presentation markers such as MHC-II. For specific induction of MHC-II –dependent T-cell activation we employed an OVA-inducible OT-II transgenic T-cell reporter system. Mature BM-DCs were treated with chicken OVA (Sigma) or OVA 323-339 fragment (AnaSpec) for 2 h at 37°C and 5% CO_2_.

#### BM-DC and OT-II T-Cell Co-incubation

OT-II T-cells were purified from spleens of OT-II transgenic mice. Single cell suspensions were generated as described above and CD4+ T-cells were separated via magnetic bead-mediated depletion of non-CD4+ cells according to manufacturer's instructions (MACS “untouched” CD4+ T-cell Isolation Kit, Miltenyi Biotec). OVA-antigen-presenting BM-DCs were co-incubated with CD4+ OT-II T-cells for 40 h at 37°C and 5% CO_2_. In order to analyze the OVA-specific T-cell response, the cell culture supernatant containing CD4+ OT-II T-cells was harvested, re-stimulated and stained for surface markers as described above for “T-cell panels.” For intracellular staining we used the following fluorophore-conjugated antibodies: eFluor 450 anti-IFNγ (clone XMG1.2, eBioscience, Thermo Scientific), PE-eFluor 610 anti-ki67 (clone SolA15, eBioscience, Thermo Scientific). T-cells were acquired and analyzed via flow cytometry. After removing the T-cell suspension, adherent layer of BM-DCs was mildly removed by incubating the cells for 10 min with 3 mM EDTA in HBSS (without Mg^2+^ and Ca^2+^) on ice. BM-DCs were analyzed via flow cytometry; we used the following fluorophore-conjugated antibodies against surface markers: PE-Cy5.5 anti-CD45 (clone 30-F11, eBioscience, Thermo Scientific), PE-Cy7 anti-CD11b (clone M1/70, eBioscience, Thermo Scientific), APC anti-CD11c (clone N418, BioLegend), PE anti-MHC class II (clone M5/114.15.2, Biolegend), FITC anti-CD80 (clone 16-10A1, eBioscience, Thermo Scientific), APC-Cy7 anti-CD86 (clone GL-1, BioLegend).

#### Oligomeric Aβ1-42 Preparation and Treatment

We used commercially available human recombinant Aβ1-42 peptide and scrambled (scr) control peptide [Beta-Amyloid (1–42), Ultra Pure, TFA and Beta-Amyloid (1–42), Scrambled, TFA; from rPeptide]. The following scrambled peptide sequence was used in all experiments: KVKGLIDGAHIGDLVYEFMDSNSAIFREGVGAGHVHVAQVEF. Throughout the experiments, Aβ1-42 peptide and scrambled peptide were processed in exactly the same way. Lyophilized peptides (1 mg vials) were reconstituted in 200 μl hexafluoroisopropanol (HFIP, Sigma), split into 20 μl aliquots (each 100 μg peptide), re-lyophilized and stored at −80°C. Oligomeric Aβ1-42 species were obtained according to established protocols (32). Stored monomeric peptide aliquots (100 μg) were reconstituted in dimethyl sulfoxide (DMSO, Gibco, Thermo Scientific) at 5 mM, sonicated for 10 min, diluted in sterile PBS (Gibco, Thermo Scientific) at 100 μM and incubated for 24 h at 4°C in an Eppendorf tube shaker (300 rpm). Bigger aggregates were excluded by centrifugation at 19,000 × g for 20 min at 4°C (Centrifuge 5417R, Eppendorf). SDS-PAGE (precast Novex 10–20% tris-glycine gels, 1.0 mm × 10 well, Invitrogen, Thermo Scientific) and silver staining according to standard protocols confirmed oligomeric state (Figure S4). For silver staining, in brief, gels were fixed for 30 min in fixing solution (40% (v/v) ethanol, 10% (v/v) acetic acid in H_2_O). Fixation was continued with fresh fixing solution for up to 18 h. Fixed gels were washed in H_2_O for 5 min and incubated in sensitizing solution (in H_2_O: 0.16 mM sodium thiosulfate, Sigma) for 2 min. After 3 washes in H_2_O, gels were treated with staining solution (in H_2_O: 2.6 mM silver nitrate and 1:100 dilution of 37% (w/v) formaldehyde solution, all chemicals from Sigma) for 35 min, followed by another 3 washes in H_2_O. Eventually, gels were placed in developer solution (in H_2_O: 30 mM sodium carbonate, 0.2 mM sodium thiosulfate and 1:240 dilution of 37% (w/v) formaldehyde solution, all chemicals from Sigma) and development was stopped after 10 min with 3% (v/v) acetic acid in H_2_O.

During antigen presentation assays, BM-DCs were treated with 2, 0.5 or 0.1 μM oligomeric Aβ1-42 or scr peptide control on day 8 of the cultivation process prior LPS-maturation. Aβ1-42 oligomer treatment of BM-DCs was started in an immature state considering Aβ oligomer-formation as initial event contributing to inflammatory activation of local brain APCs. A replenishment of Aβ1-42 oligomers followed in parallel with co-incubation of OT-II T-cells.

#### Amylin Preparation and Treatment

Aggregated Amylin controls were produced using human synthetic Amylin1-37 (Amylin (human) trifluoroacetate salt, Bachem). Aggregation was achieved by incubation for 24 h at 37°C in an Eppendorf tube shaker (300 rpm). Low-molecular aggregation state was confirmed by SDS-PAGE and silver staining according to standard protocols. Amylin treatment was carried out as described for oligomeric Aβ1-42.

### RNA Isolation

Total RNA was extracted using the GenElute Mammalian TotalRNA Kit (Sigma). The RNA concentration was measured using RNA HS Assay kit (Thermo Fisher Scientific) and RNA integrity was analyzed with the Qubit RNA IQ Assay (Thermo Fisher Scientific).

### RNA Sequencing (RNAseq)

*RNA sequencing service* was provided by the Next Generation Sequencing (NGS) platform at the University of Bern, Switzerland.

#### Library Preparation and Sequencing

A library of mRNA sense transcripts was prepared using the TruSeq Stranded mRNA kit (Illumina). RNAseq was performed in single reads of 100bp length using Illumina HiSeq3000 (Illumina). The raw sequence data were uploaded to the public repository NCBI-GEO and are available via accession number *GSE136789*.

#### Quality Control and Data Analysis

After quality control of RNAseq data (for more detail see Supplementary Material and Methods), the reads were mapped to the mouse reference genome. We tested for differential gene expression between the experimental groups. The log-fold change of each gene was adjusted to include the evidence based on which the log-fold change was estimated and to extrapolate the adjusted *p*-value (adjp). Gene ontology (GO) enrichment analysis was performed to identify gene sets containing differentially expressed genes. Genes were ranked within a GO term by comparing the proportion of differentially expressed genes among all genes assigned to the GO term with all other genes, and by sorting all genes by *p*-value as well as testing if the genes assigned to a particular GO term are enriched at the top or the bottom of this ranking. All analyses were run in R (version 3.4.4). The top 10 GO terms of each category (Biological Process, Cellular Compartment and Molecular Function) for each BM-DC treatment comparison were listed based on the assigned *p*-value. The overall 30 top GO hits from each comparison were grouped using a Venn diagram to identify GO terms specifically enriched in each individual BM-DC treatment comparison.

#### Reverse Transcription and Quantitative Real-Time PCR

Selected genes of interest were analyzed for their relative expression changes using quantitative Real-Time PCR (qPCR). First, reverse transcription was performed in 20 μl reaction mixture containing 1 μg of RNA, 1x PCR buffer, 5 mM MgCl2, 10 mM of each dNTP, 0.625 μM oligo dT_16_, 1.875 μM random hexamers, 20 U RNase inhibitor and 50 U MuLV reverse transcriptase (Life Technologies). The cycles for the reverse transcription were set as follows: 25°C for 10 min, 42°C for 1 h, followed by 99°C for 5 min. The resulting cDNA was amplified in duplicate by qPCR in 10 μl reaction mixture with 200 nM of each specific primer (Table S14) and 1x Fast Syber Green qPCR MasterMix (Life Technologies). The amplification reaction was performed with QuantStudio 7 Flex (Applied Biosystems, Life Technologies). The amplification program was set as follows: 95°C for 5 min, followed by 40 cycles at 95°C for 10 s, 60°C for 15 s, 72°C for 20 s. *Gapdh* and *Rplp2* served as housekeeping genes and their amplification data were averaged and used for sample normalization. Excel (Microsoft Office) was used for the comparative quantification analysis.

### Statistical Analysis

We used GraphPad Prism analysis software for statistical testing (Version 8.21, GraphPad Software). We applied one-way ANOVA with Bonferroni's *post hoc* test for comparison of more than two experimental groups. We performed two-way ANOVA with Bonferroni's *post hoc* test for multiple comparisons of experimental groups influenced by two different independent variables. Data are shown as mean ± standard deviation (SD). For Pearson's correlation we calculated Pearson coefficient r and tested the *p*-value via two-tailed testing. For qPCR data, we applied multiple *t*-test analysis with Holm-Sidak *post hoc* test. Significance was considered at *p* < 0.05 if not otherwise specified.

## Results

### APP-PS1 Mice Show Predictable and Time-Dependent Plaque and Aβ-Oligomer Accumulation

**Figure 1:**
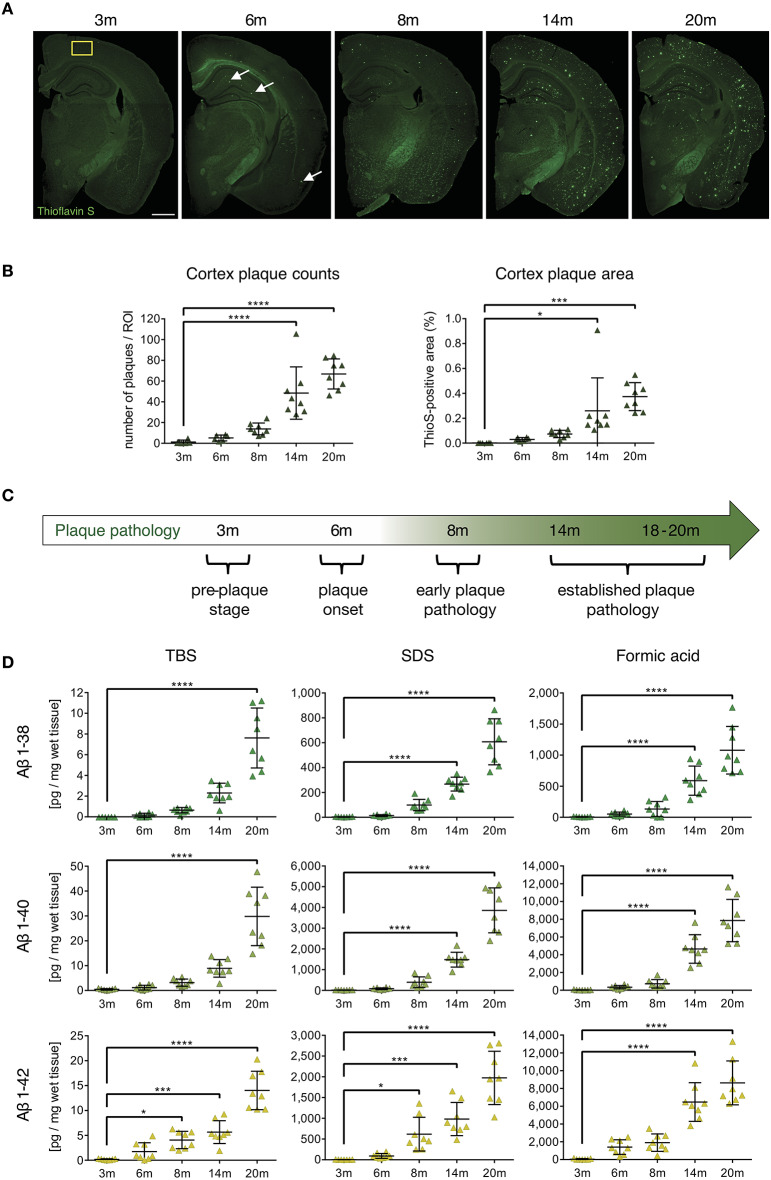
Characterization of cerebral plaque load and Aβ species in the APP-PS1 transgenic mouse model. **(A)** Representative images of Thioflavin-S staining of coronal sections from APP-PS1 transgenic (tg) mice at different ages. First occasional plaques are observed at 6 months of age in cortex and anterior hippocampus (white arrows). Diffuse staining in corpus callosum and other white matter bundles is non-specific, as it is observed in non-tg mice as well. Scale bar = 0.8 mm. Yellow box (0.33 mm^2^) shows area used for quantification. **(B)** Quantitative analysis of plaque load in the cortex shows progressively increased beta-amyloid plaque numbers with age. Only significant differences between pre-plaque stage (3 m) and other plaque pathology stages are shown. **(C)** Schematic of identified stages of beta-amyloid pathology progression in APP-PS1 tg mice. **(D)** MSD-analysis of sequential protein extracts from prefrontal cortex reveals that TBS and SDS-soluble Aβ1-42 species start to accumulate already at 8 months of age. Each symbol represents data from one tg mouse. Only significant differences between pre-plaque stage (3 m) and other plaque pathology stages are shown. 3 m, *n* = 8; 6 m, *n* = 8; 8 m, *n* = 8; 14 m, *n* = 8; 20 m, *n* = 8. (Data are shown as mean ± SD, *p < 0.05, ***p < 0.001, ****p < 0.0001, one-way ANOVA with Bonferroni's multiple comparisons test).

In order to analyze the impact of beta-amyloid pathology on APCs and T-cells, we first identified and characterized specific stages of beta-amyloid disease progression in the APP-PS1 transgenic mouse model in our hands. Quantitative analysis of Thioflavin-S stained brain sections from cortex (Figure 1A) and anterior hippocampus (Figure S1) in terms of plaque load revealed no detectable beta-amyloid deposits at 3 months of age. Initial beta-amyloid deposition at 6 and 8 months of age was followed by established plaque pathology in brains of 14- and 20-months old APP-PS1 transgenic animals (Figure 1B, detailed list of *p*-values in Tables S1–S4). Therefore, for the subsequent analyses we defined the following pathology stages: pre-plaque stage (3-months old), plaque onset stage (6-months old), early plaque pathology (8-months old) and established plaque pathology (14- and 18–20-months old) (Figure 1C). Since oligomers of Aβ have been shown to be the most neurotoxic species and can occur also before overt plaque deposition (33), we applied MSD assay to analyze the oligomeric Aβ content of protein extracts from prefrontal cortex of APP-PS1 transgenic mice at different disease stages (Figure 1D, detailed list of *p*-values in Tables S5–S13). Indeed, both TBS-soluble and SDS-soluble Aβ1-42 fractions (which are enriched in Aβ oligomers) increased significantly already at 8 months of age.

These results indicate that neurotoxic oligomeric Aβ1-42 species start accumulating in the APP-PS1 model already at early plaque pathology stage. Having defined the temporal progression of beta-amyloid pathology in transgenic mice, we proceeded to examine which beta-amyloid variant would elicit APC and T-cell alterations.

### Reduction of MHC-II Surface Expression on CNS APCs Coincides With Early Plaque Pathology

In previous studies we found decreased surface expression of MHC-II molecules on brain APCs in both the APP-PS1 and the ArcAβ transgenic mouse model at 24 months of age (27), representing an established plaque pathology stage. In those studies, however, the exact onset of APC alterations during beta-amyloid pathology progression was not addressed. In particular, it was not clear, whether the alterations were late events, possibly secondary to other pathological processes, or if they were elicited by specific Aβ aggregation states.

**Figure 2:**
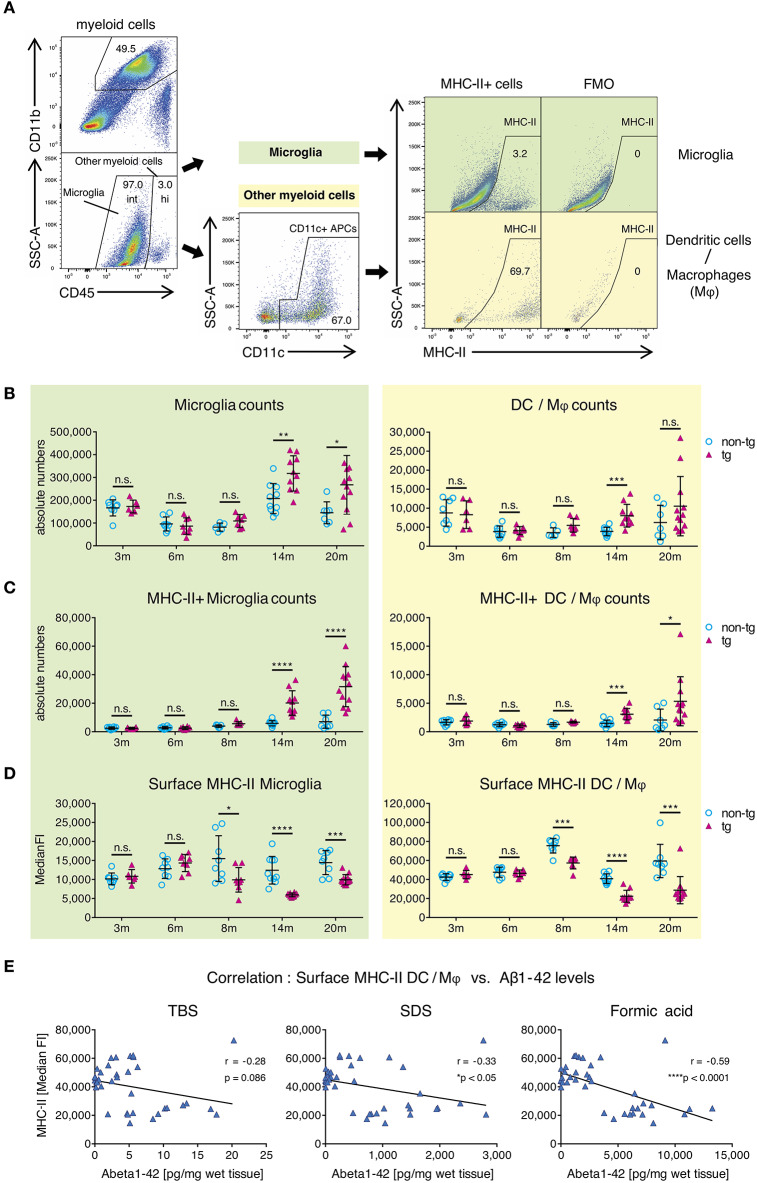
Alterations in MHC class II surface expression on APCs in brains of APP-PS1 transgenic mice. **(A)** Schematic of flow cytometry analysis of cerebral APCs. Myeloid cells (CD45+, CD11b+) are gated based on CD45 levels to distinguish “microglia” (CD45 intermediate, int) and “other myeloid cells” (CD45 high, hi). Dendritic cells (DC) and CNS border-associated macrophages (Mφ) are gated from “other myeloid cells” based on CD11c expression (= DC/Mφ). FMO = fluorescence minus one control. Numbers in gates indicate percent cells of parental gate. **(B)** Absolute numbers of microglia and DC/Mφ are elevated in brains of APP-PS1 tg mice at established plaque pathology stages (14 and 20 months of age) compared to non-tg, age-matched animals. **(C)** Analysis of surface MHC class II (MHC-II) expression on microglia and DC/Mφ. Numbers of MHC-II+ microglia and DC/Mφ are increased in tg brains starting at 14 months of age. **(D)** In contrast, surface MHC-II expression per cell (measured by median fluorescence intensity = MedianFI), is reduced in cells derived from tg animals, starting at 8 months of age in both microglia and DC/Mφ. Each symbol represents data from one mouse. 3 m, *n* = 8 (non-tg), *n* = 6 (tg); 6 m, *n* = 8 (non-tg), *n* = 8 (tg); 8 m, *n* = 5 (non-tg), *n* = 7 (tg); 14 m, *n* = 10 (non-tg), *n* = 10 (tg); 20 m, *n* = 7 (non-tg), *n* = 12 (tg). (Data are shown as mean ± SD, *p < 0.05, **p < 0.01, ***p < 0.001, ****p < 0.0001, n.s. = non-significant, two-way ANOVA with Bonferroni's multiple comparisons test). **(E)** Correlation of Aβ1-42 species with cerebral MHC-II surface levels. Insoluble amyloidogenic Aβ species significantly correlate with reduced MHC-II surface expression on DC/Mφ. Each symbol represents data from one tg mouse (*n* = 37). Each graph uses the same data on surface MHC-II (in MedianFI) in correlation with respective Aβ species concentration (Pearson's correlation, Pearson coefficient r indicates negative correlation, two-tailed p-value testing).

To clarify this, we used flow cytometry to analyze surface MHC-II levels on CNS-resident APCs, including leptomeningeal and choroid plexus DCs, CNS border-associated macrophages (Mφ) and microglia in APP-PS1 transgenic mice. Myeloid cells from mononuclear cell extracts were identified via CD45 and CD11b expression (Figure 2A) and further differentiated into microglia with intermediate (int) CD45 expression (CD45int CD11b+) and CD45 high (hi) other myeloid cells (CD45hi CD11b+) (18). CD45hi CD11b+ myeloid cells were additionally gated for CD11c, in order to select *bona fide* DCs and Mφ with potent T-cell-stimulating activity. Several microglia subsets can express CD11c as well (34). However, the functional role of this marker in microglia is not fully understood, thus we did not gate for CD11c+ microglia cells.

By analyzing microglia and DC/M φ numbers and MHC-II expression via flow cytometry, we observed significantly higher absolute numbers of all APCs in brains of transgenic APP-PS1 animals with established plaque pathology (14- and 20-months old) compared to age- and sex-matched non-transgenic littermates (Figure 2B). This was paralleled by an increase in numbers of MHC-II+ APCs in transgenic brains, starting at 14 months of age (Figure 2C). In non-transgenic brains, only a few MHC-II+ APCs were observed; we have previously shown that these cells tend to localize in perivascular spaces, meninges, and choroid plexus (27).

In order to analyze MHC-II surface expression levels per cell we examined the median fluorescence intensity (MedianFI) of MHC-II (Figure 2D). Notably, DC/Mφ, even though existing in small numbers, showed higher levels (about 4x higher in average) of baseline surface MHC-II expression per cell compared to microglia, as expected based on their more efficient antigen presentation capability. However, in contrast to the overall increasing absolute number of cerebral MHC-II+ cells in beta-amyloid pathology-burdened brains, we observed a reduction in surface MHC-II expression per cell on microglia and DC/Mφ in APP-PS1 transgenic mice. This effect was already observed at 8 months of age, corresponding to the earliest accumulation of oligomeric Aβ1-42 species (Figure 1D). Indeed, an inverse and significant correlation was found between the MedianFI levels of MHC-II and highly amyloidogenic SDS- and formic acid-soluble Aβ1-42 species, but not TBS-soluble forms. Mice having the highest levels of aggregated Aβ1-42 had the lowest expression of surface MHC-II on the surface of their APCs (Figure 2E, Figure S2A). No effects on surface MHC-II were seen in spleen-derived DCs from APP-PS1 transgenic animals (Figure S2B), suggesting a brain-specific effect.

These results suggest that the appearance of amyloidogenic oligomeric Aβ1-42 species affects the numbers and phenotype of brain APCs.

### Pro-Inflammatory Effector T-Cell Response Is Reduced in Late-Stage Plaque Pathology

**Figure 3:**
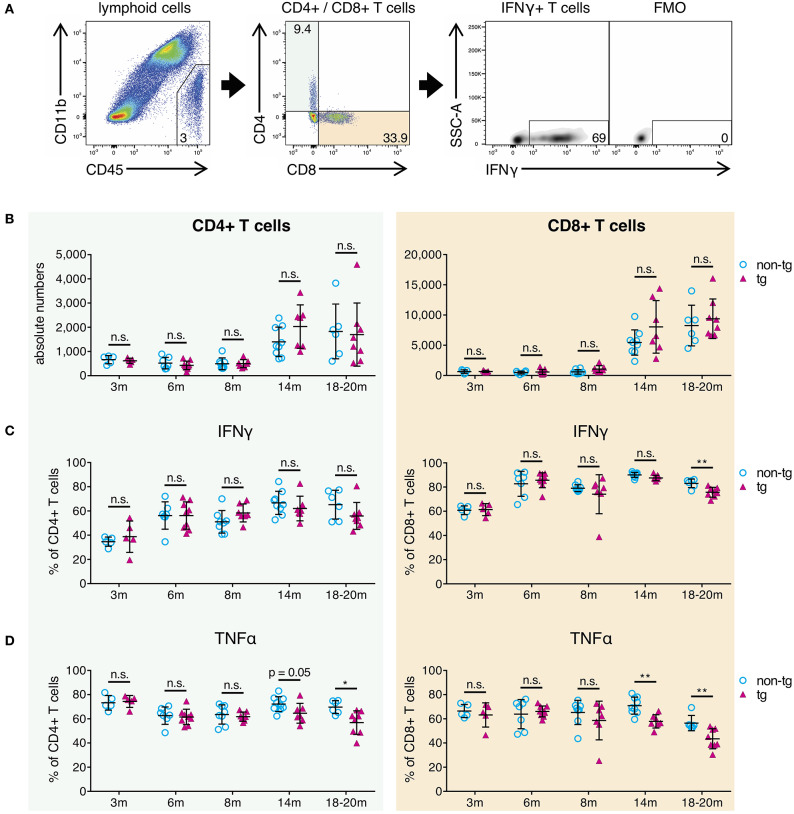
Decrease of pro-inflammatory CD4+ and CD8+ effector T-cells in brains of aged APP-PS1 transgenic mice. **(A)** Schematic of flow cytometry analysis of cerebral lymphoid cells derived from APP-PS1 mouse brains. T-cells (TCRβ+) are identified in lymphoid cell population (CD45+, CD11b-) and subdivided into CD4+ T-helper cells and CD8+ cytotoxic T-cells. FMO = fluorescence minus one control. Numbers in gates and quadrants indicate percent cells of parental gate. **(B)** Baseline numbers of CD4+ and CD8+ T-cells increase during aging. However, no difference is observed between non-tg and APP-PS1 tg animals. **(C,D)** Flow cytometry analysis of intracellular pro-inflammatory IFNγ- and TNFα-content reveals **(C)** decreased frequency of IFNγ-producing CD8+ T-cells at 18–20 months of age and **(D)** reduced frequency of TNFα-secreting CD4+ and CD8+ T-cells starting at 14 months of age in APP-PS1 tg animals. Each symbol represents data from one mouse. 3 m, *n* = 5 (non-tg), *n* = 5 (tg); 6 m, *n* = 7 (non-tg), *n* = 9 (tg); 8 m, *n* = 8 (non-tg), *n* = 7 (tg); 14 m, *n* = 9 (non-tg), *n* = 7 (tg); 18-20 m, *n* = 6 (non-tg), *n* = 8 (tg). (Data are shown as mean ± SD, *p < 0.05, **p < 0.01, n.s. = non-significant, two-way ANOVA with Bonferroni's multiple comparisons test).

Next, we analyzed whole-brain T-cells from all pathology stages of APP-PS1 transgenic mice via flow cytometry in terms of absolute numbers and effector responses. We selected all non-myeloid cells (CD45+ CD11b-), used a marker against the T-cell receptor beta chain (TCRβ) as T-cell identifier and characterized CD4+ helper T-cells and cytotoxic CD8+ T-cells separately (Figure 3A).

In this study, absolute CD4+ and CD8+ T-cell numbers in brains of APP-PS1 transgenic mice did not diverge compared to non-transgenic littermates in any disease stage of this model (Figure 3B). As also previously shown, CD8+ T-cells are the predominant T-cell population observed in brains, and baseline numbers of CD4+ and CD8+ T-cells are increased in aged mouse brains (27). Moreover, we have already reported that CNS-infiltrating CD8+ T cells in APP-transgenic mice do not specifically co-localize with either neurons, microglia or astrocytes, and are only loosely related to beta-amyloid plaques (27).

The expression of signature cytokines was used to define specific T-cell subtypes. For the pro-inflammatory T-cell spectrum we analyzed vesicle-stored cytokines such as interferon gamma (IFNγ) and tumor necrosis factor alpha (TNFα) via flow cytometry. Furthermore, in order to monitor tolerogenic T-cell phenotypes, we used interleukin-10 (IL-10) as signature cytokine of anti-inflammatory Th2-cells and transcription factor FoxP3 for regulatory T-cells. While tolerogenic T-cells remained unchanged (Figure S3A), we observed alterations in pro-inflammatory IFNγ- and TNFα-expressing T-cells in established plaque pathology stages for APP-PS1 transgenic mice. We noticed a reduced frequency of IFNγ-producing CD8+ T-cells at 18–20 months of age (Figure 3C) and a decrease in the proportion of TNFα-secreting CD4+ and CD8+ T-cells starting at 14 months of age in APP-PS1 transgenic animals (Figure 3D). None of these effects were observed in inguinal lymph node-derived CD4+ or CD8+ T-cells (Figure S3B), suggesting a brain-specific, amyloid precursor protein (APP)- and presinilin-1 (PS1)-independent effect.

Taken together, our results confirm that the frequency of pro-inflammatory IFNγ/TNFα-expressing effector T-cells is reduced in brains with established beta-amyloid pathology. Since this occurred months after the first observed alterations of APCs at 8 months of age, impaired T-cell responses might be secondary to APCs' inhibition.

### Aβ1-42 Oligomers Inhibit Antigen-Specific CD4+ T-Cell Responses *in vitro*

**Figure 4:**
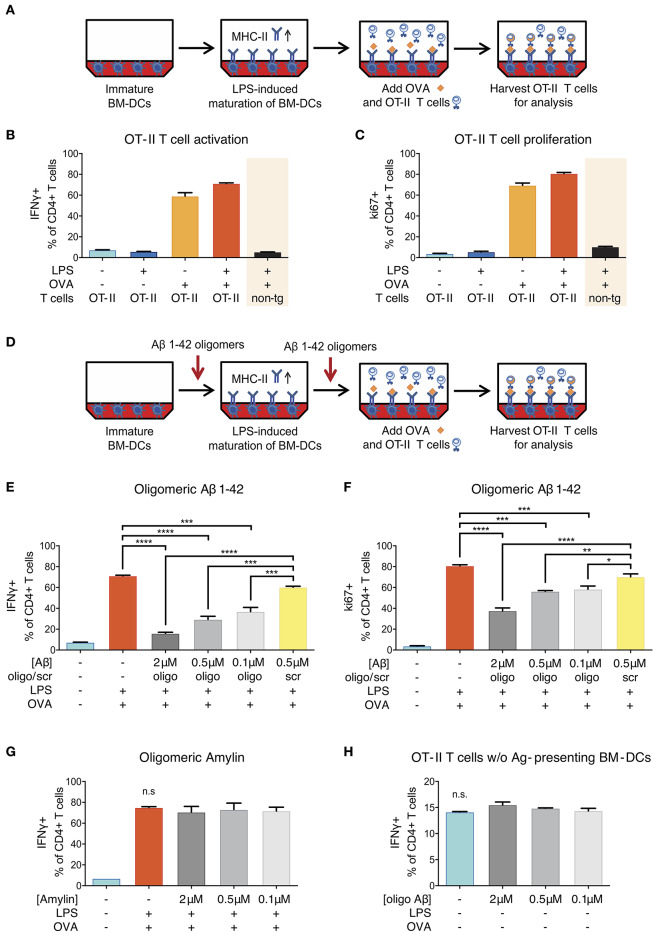
Reduction of ovalbumin (OVA)-specific OT-II T-cell response following Aβ1-42 oligomer treatment. **(A)** Schematic of *in vitro* antigen presentation assays. LPS-matured bone marrow-derived dendritic cells (BM-DCs) process full-length ovalbumin (OVA) and present antigenic material. OVA-specific OT-II tg CD4+ T-cells are added to the BM-DCs and co-incubated for 40 h. IFNγ and ki67 are analyzed in harvested T-cells via flow cytometry as markers of T-cell activation and proliferation, respectively. Full activation (more IFNγ+ cells) **(B)** and proliferation (more ki67+ cells) **(C)** of T-cells specifically requires the presence of mature BM-DCs (treated with LPS) and OVA-derived antigens. T-cell activation is antigen-specific and does not occur in the absence of OVA, nor if non-tg CD4+ T-cells are used instead of OVA-specific OT-II CD4+ T-cells. **(D)** The effect of Aβ is tested by incubating immature BM-DCs with human recombinant Aβ1-42 (oligomeric state) or scrambled (scr) control. **(E,F)** Recombinant Aβ1-42 oligomers, but not scr peptide, significantly inhibit T-cell activation and proliferation by reducing the frequency of IFNγ+ **(E)** and ki67+ **(F)** OT-II CD4+ T-cells in a dose-dependent manner. **(G)** Human aggregated Amylin1-37 has no effect on IFNγ response. **(H)** Incubation of OT-II CD4+ T-cells with Aβ1-42 oligomers but without antigen-presenting BM-DCs does not affect IFNγ response. Representative results of at least 3 independent experiments are shown in each graph; *n* = 2 per treatment condition in each experiment. (Data are shown as mean ± SD, *p < 0.05, **p < 0.01, ***p < 0.001, ****p < 0.0001, n.s. = non-significant, one-way ANOVA).

To test whether oligomeric forms of Aβ1-42 could cause direct and acute impairment of antigen presentation, we took advantage of a well-established *in vitro* model (31) using lipopolysaccharide (LPS)-matured murine primary bone marrow-derived dendritic cells (BM-DCs) as prototypical APCs instead of cerebral microglia and DC/Mφ. In this assay, full-length chicken ovalbumin (OVA) was supplied as antigen, phagocytosed by BM-DCs, processed intracellularly and presented on their surface via MHC-II. In combination with OVA-specific OT-II transgenic CD4+ T-cells as responders we set up inducible antigen presentation assays (Figure 4A).

Successful antigen presentation of OVA toward OT-II CD4+ T-cells was measured by analyzing intracellular IFNγ production (as marker of T-cell activation) and intranuclear ki67 expression (as marker of T-cell proliferation) via flow cytometry. In the presence of LPS-matured BM-DCs, we observed an expected increase in the frequency of IFNγ- and ki67-producing T-cells (Figures 4B,C). T-cell activation and proliferation was antigen-specific and did not occur in the absence of OVA, nor if non-transgenic CD4+ T-cells were used instead of OVA-specific OT-II CD4+ T-cells.

In order to test the effect of Aβ on antigen presentation, we added a preparation of aggregated human recombinant Aβ1-42 containing various forms of oligomers (Figure S4) in doses ranging from 0.1 to 2 μM to the antigen presentation assays (Figure 4D). This dose range was chosen as it mimics the Aβ oligomer concentration reported in human AD brains (35) and is widely used in Aβ peptide-related *in vitro* studies (36). We found that Aβ1-42 oligomers significantly reduced the frequency of IFNγ+ (Figure 4E) as well as ki67+ OT-II CD4+ T-cells (Figure 4F) as compared to untreated control cells and cells treated with non-aggregating scrambled (scr) control peptides. These inhibitory effects on T-cell activation and proliferation were dose-dependent and not observed with scr control peptide.

### T-Cell Inhibition Is Driven by Beta-Amyloid-Specific Effects on Antigen-Presenting BM-DCs

Having observed an inhibitory effect of Aβ on the induction of T-cell activation and proliferation in the context of *in vitro* antigen presentation, we aimed at characterizing the mechanisms. First, we investigated whether the effect was Aβ1-42 sequence-dependent, or simply due to amyloidogenic capacity of a peptide. We therefore applied human Amylin1-37, the major component of protein deposits found in the islets of Langerhans in patients with noninsulin-dependent diabetes mellitus. Aggregated Amylin1-37 at different doses (2, 0.5, 0.1 μM) had no influence on IFNγ response (Figure 4G) nor ki67 expression (Figure S5A) of OT-II CD4+ T-cells. Based on those results, we conclude that the effect of Aβ1-42 oligomers on APCs is peptide sequence-specific.

Second, we tested whether the reduced activation of OT-II CD4+ T-cells in our assays with Aβ1-42 oligomers was due to a direct effect on T-cells. To study this, we incubated OT-II CD4+ T-cells with Aβ1-42 oligomers without antigen-presenting BM-DCs and analyzed activation and proliferation status of re-stimulated T-cells. The frequency of IFNγ- (Figure 4H) and ki67-producing cells (Figure S5B) were unchanged following Aβ1-42 oligomer treatment. Furthermore, the apoptosis-marker AnnexinV in combination with a dead cell staining (“Zombie”) revealed no overt toxic effect of Aβ1-42 oligomers on T-cells (Figure S5G). These results indicate that Aβ1-42 oligomers might not act on T-cells directly, but most probably exert their action on antigen-presenting BM-DCs.

### Inhibitory Effects of Human Oligomeric Beta-Amyloid on APCs Are Independent of Antigen-Processing and -Presenting Machinery

After exclusion of direct effects on T-cells, we focused on Aβ1-42-mediated effects on BM-DCs to investigate the mechanisms responsible for reduced antigen presentation. First, we analyzed the viability of Aβ1-42 oligomer-treated BM-DCs. No changes in viability were observed for BM-DCs treated with 2 or 0.5 μM Aβ1-42 oligomers compared to cells treated with 0.5 μM scr peptide or untreated mature BM-DCs (Figure S5H), suggesting a specific action of Aβ1-42 oligomers on BM-DC function.

**Figure 5:**
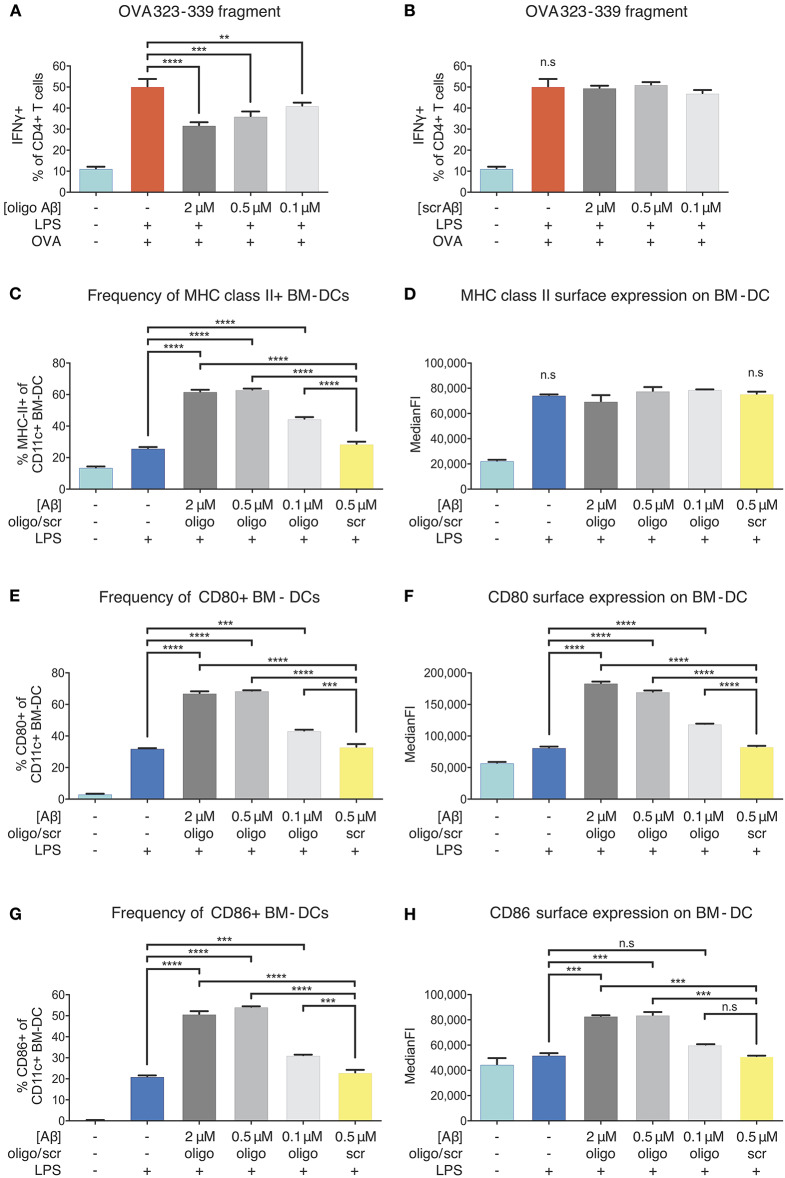
Inhibition of APCs by Aβ1-42 oligomers *in vitro* without involvement of antigen-processing and -presenting apparatus. The effect of Aβ1-42 oligomers on antigen-processing was tested by using OVA 323-339, a fragment that leads to robust OT-II T-cell response without intracellular processing. Addition of Aβ1-42 oligomers **(A)**, but not scrambled (scr) peptide **(B)**, inhibits T-cell activation in a dose-dependent manner, indicating that inhibitory effects of Aβ1-42 oligomers are independent of APCs' antigen-processing machinery. **(C)** Flow cytometry analysis of surface MHC-II expression shows that Aβ1-42 oligomers (but not scr peptide) increase MHC-II+ BM-DC frequencies. **(D)** In comparison with LPS-matured BM-DCs, no changes in surface MHC-II expression per cell (median fluorescent intensity = MedianFI) is detectable after applying Aβ1-42 oligomers to *in vitro* antigen presentation assays. **(E,G)** Analysis of surface co-stimulatory factor CD80/CD86 expression shows that Aβ1-42 oligomers (but not scr peptide) increase CD80+/CD86+ BM-DC frequencies. **(F,H)** In comparison with LPS-matured BM-DCs, surface expression of CD80/CD86 per cell (measured by MedianFI) is further increased after applying Aβ1-42 oligomers. Representative results of at least 3 independent experiments are shown in each graph; *n* ≥ 2 per treatment condition in each experiment. (Data are shown as mean ± SD, **p < 0.01, ***p < 0.001, ****p < 0.0001, n.s. = non-significant, one-way ANOVA).

Next, we aimed at defining the exact stage at which antigen presentation is affected. In fact, antigen presentation on MHC-II surface molecules is preceded by a series of events including phagocytosis of antigens, enzymatic digestion and peptide fragmentation in phagolysosomal/late endosomal compartments, as well as MHC-II loading mechanisms and transport for stable integration into the plasma membrane (37). We investigated whether the inhibitory effect of Aβ1-42 oligomers on APCs was due to an interference with the intracellular antigen-processing machinery of BM-DCs. To test this, we repeated the assay using OVA *323-339* fragment, a small antigenic peptide fragment of OVA that contains the CD4+ T-cell epitope. This fragment binds directly to surface MHC-II on APCs, without being processed intracellularly. Indeed, OVA *323-339* fragment sparked similar antigen-specific and inducible OT-II T-cell activation and proliferation responses (Figures S5C,D) as seen for full-length OVA treatment. The addition of different doses of Aβ1-42 oligomers (2, 0.5, 0.1 μM) (Figure 5A), but not scr peptide (Figure 5B), decreased the frequency of IFNγ-producing OT-II CD4+ T-cells in a dose-dependent manner. Similar results were observed for ki67+ OT-II CD4+ T-cells (Figures S5E,F).

T-cell responses to OVA *323-339* fragment are dependent on pre-existing surface MHC-II molecules (38) (Figures S5C,D), since intracellular processing and MHC-II loading are circumvented. As oligomeric Aβ was associated with reduced levels of surface MHC-II in our *ex vivo* experiments, we aimed at testing whether Aβ would alter MHC-II expression on BM-DCs. Flow cytometric examination confirmed that LPS maturation induced a significant increase in the frequency of MHC-II+ BM-DCs and surface expression of MHC-II, as expected. We found that Aβ1-42 oligomer treatment (but not scr peptide) further elevated the frequency of MHC-II+ BM-DCs, with no effect on MHC-II surface expression, as compared to only LPS-stimulated cells (Figures 5C,D).

Since secondary signals provided by co-stimulatory surface molecules are required to induce effective T-cell priming, we further analyzed CD80 and CD86 expression on BM-DCs. Treatment with Aβ1-42 oligomers (but not scr peptide) increased CD80+/CD86+ BM-DC frequencies (Figures 5E,G) and surface expression of CD80 and CD86 compared to only LPS-stimulated cells (Figures 5F,H).

Thus, reduced antigen presentation *in vitro* following Aβ1-42 oligomer exposure cannot be ascribed to interference with intracellular antigen processing nor to a reduction of surface MHC-II or co-stimulatory factors. These findings suggest that more complex mechanisms underlie beta-amyloid-induced reduction of antigen presentation by APCs.

### Aβ1-42 Oligomers Alter Gene Expression of Key Immune Mediators in BM-DCs

**Figure 6:**
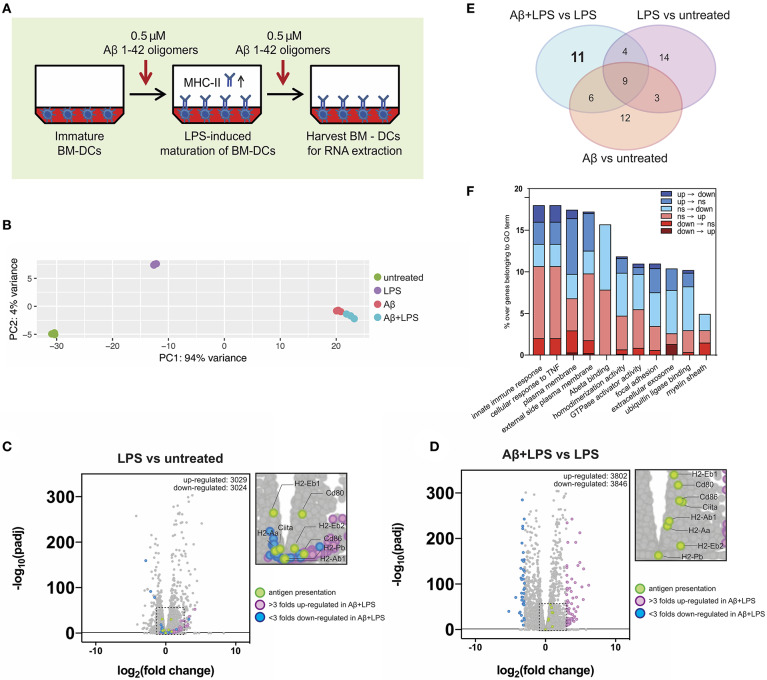
Gene expression alterations of key immune factors in dendritic cells following Aβ1-42 oligomer treatment. **(A)** Schematic of *in vitro* treatment of mouse BM-DCs with 0.5 μM Aβ1-42 oligomers for 4 days in total, starting 1 day before LPS-induced maturation (“Aβ+LPS” full setup). BM-DCs are harvested for RNA-extraction and -sequencing. **(B)** Principal component analysis (PCA) of RNAseq data. Four conditions are tested: untreated BM-DCs (“Untreated”; *n* = 3), LPS stimulation only (“LPS”; *n* = 3), Aβ stimulation only (“Aβ”; *n* = 3) and Aβ stimulation combined with LPS treatment (“Aβ+LPS”; *n* = 3). **(C,D)** Volcano plots of RNAseq data for “LPS vs. Untreated” **(C)** and “Aβ+LPS vs. LPS” **(D)** comparisons. Key genes involved in antigen presentation are highlighted in green. Genes that are either more than 3 folds up-regulated (violet) or down-regulated (blue) in “Aβ+LPS vs. LPS” are highlighted. Significance is set at adjp < 0.05. **(E)** Venn diagram groups GO terms that are specifically enriched in each individual BM-DC treatment comparison. 11 GO-terms are exclusively altered by Aβ treatment of LPS-matured BM-DCs. **(F)** Aβ-induced gene expression changes within each of the 11 considered GO terms compared to LPS-maturation only (“Aβ+LPS vs. LPS” compared to “LPS vs. Untreated”). Bar graph depicts ratio of genes affected by the presence of Aβ over the total number of genes in the respective GO term. Gene expression switches are color-coded and defined as follows: (i) up-regulated (“up”; fold change > 1; adjp ≤ 0.05), (ii) down-regulated (“down”; fold change < −1; adjp ≤ 0.05) and (iii) non-significant (“ns”; −1 ≤ fold change ≤ 1; adjp > 0.05).

Our *in vitro* setup of antigen-presenting BM-DCs and T-cells showed that antigen presentation was impaired in BM-DCs treated with Aβ1-42 oligomers, but expression of surface MHC-II and related co-stimulatory factors remained intact. Hence, we speculated that aggregated Aβ might affect BM-DC gene expression of single genes or entire gene sets that might be involved in antigen presentation or mediate the observed inhibitory effect. To obtain an unbiased view of gene regulation following Aβ1-42 oligomer treatment, we set up antigen presentation assays as described previously (Figure 6A) and examined BM-DC gene transcription via explorative RNA sequencing. We analyzed the transcriptome of untreated immature control BM-DCs (“Ctrl”), LPS-treated BM-DCs (“LPS”), Aβ1-42 oligomer-treated BM-DCs (“Aβ”) and BM-DCs treated with Aβ1-42 oligomers and LPS (“Aβ+LPS”).

First, we used principal component analysis (PCA) to visualize the 500 most variable genes of each experimental group for similarity assessment (Figure 6B). Triplicates from the same experimental group clustered in close proximity in the PCA plot. As expected, LPS stimulation shifted BM-DC gene expression; however, both Aβ-treated groups (“Aβ” and “Aβ+LPS”), clustered separately from “Ctrl” and “LPS,” with much higher variance. This result indicated a strong effect of Aβ on transcriptional regulation of DCs, which differs dramatically from that of LPS.

To further confirm the robustness of our assay, we checked the expression changes of antigen presentation genes following LPS-activation (Figure 6C). Antigen-presenting MHC-II is a subject of tight transcriptional and post-translational regulation. MHC-II gene transcription is controlled by a master regulator, called “class II transactivator” (CIITA) (39). In DCs, CIITA as well as MHC-II gene transcription itself are down-regulated following maturation by LPS. Nevertheless, MHC-II protein surface levels rise upon LPS-maturation (as shown in Figure 5D), since MHC-II protein degradation is shut down as well in order to maintain long-lived MHC-II/peptide complexes on the surface (40–42). As expected, LPS-treated BM-DCs, in comparison to “Ctrl” cells, presented with down-regulation of *Ciita* [log2(fold change) = −0.42, adjp = 6.44 × 10^−6^] and MHC II-related genes such as *H2-Eb1* [log2(fc) = −0.51, adjp = 1.17 × 10^−31^], while co-stimulatory factors such as *Cd80* were up-regulated [log2(fc) = +0.77, adjp = 5.83 × 10^−31^].

Next, we examined the effect of Aβ1-42 oligomer treatment on LPS-matured BM-DCs in terms of antigen presentation-related gene transcription. By comparing “Aβ+LPS” vs. “LPS” samples (Figure 6D), we found up-regulation of *Ciita* [log2(fc) = +1.07, adjp = 1.41 x 10^−37^], MHC II-related genes such as *H2-Ab1* [log2(fc) = +0.48, adjp = 3.52 × 10^−24^] and *H2-Eb1* [log2(fc) = +0.69, adjp = 3.01 × 10^−56^], as well as co-stimulatory factors *Cd80* [log2(fc) = +0.88, adjp = 4.03 × 10^−49^] and *Cd86* [log2(fc) = +0.95, adjp = 3.15 × 10^−38^]. Those results confirmed our previous findings of higher cell numbers with surface MHC-II and co-stimulatory factor expression after Aβ treatment *in vitro*. Nevertheless, these Aβ-treated BM-DCs present with reduced antigen presentation capacity.

In order to dissect alternative Aβ-affected pathways we performed gene ontology (GO) enrichment analysis to detect sets of genes that were differentially regulated. The resulting GO terms were categorized in 3 domains: molecular function, cellular component and biological process. We identified the top 10 GO terms for every gene expression comparison within each of the 3 categories (“Aβ+LPS” vs. “LPS” GO term ranks are depicted in Figure S6). Interestingly, amongst biological processes, treatment with Aβ induced variance in the expression of immune-related genes, such as “cellular response to TNF” and “innate immune response,” indicating that Aβ can profoundly affect immune signaling of DCs. By comparing GO terms across experimental groups, we found 11 GO terms that were specifically and exclusively altered by Aβ treatment of LPS-matured DCs, and did not appear in any other experimental group (Figure 6E, detailed volcano plots in Figure S7). We applied a consecutive strategy for GO term analysis (Figure S8A) to further characterize these 11 GO terms by calculating the percentage of genes that significantly changed expression profile (“switchers”) in presence of Aβ compared to LPS-maturation only (Figure 6F). Considering switched up-regulation, down-regulation and non-significant changes, we observed that up to 18% of genes of interest were affected by Aβ within GO terms. Immune pathways such as “innate immune response” and “cellular response to TNF” were highly represented amongst the Aβ-specific gene expression changes. Within these GO terms, 2% of the genes of interest (GOI, 150 genes in total) switched completely expression profile from up- to down-regulation (including *Ccl8, Card14*, and *Tnfsf18*). Furthermore, 2.7% of GOI switched from non-significant changes during LPS-maturation to down-regulation upon Aβ treatment (e.g., *Ccl2* and *Ccl7*). Another strongly Aβ-affected GO-term was “Plasma membrane”-related GOI (1840 genes in total), which showed a total change of 17.4% following Aβ treatment compared to gene expression after LPS-induced maturation. We observed 1% of GOI switching from up- to down-regulation (including *Tnfsf18, Bdkrb2, Ltk*, and *Fyb2*) and 0.3% switching from down- to up-regulation (including *Fn1, Ednrb, CD226*, and *Tmc3*).

To confirm the results, we selected 11 switcher genes and verified their relative changes via regular qPCR. With the exception of Fyb2, we could confirm the direction of the switch for all GOI. Importantly, we found a significant down-regulation of key immune modulators such as CCL8 and TNFSF18 (Figure S8B). These results suggest that the prerequisites for proper antigen presentation might be impaired on a transcriptional level due to changes in immune pathways following Aβ exposure in this *in vitro* model.

## Discussion

Our data on antigen presentation inhibition in the presence of aggregated Aβ species adds to the mounting body of genetic and experimental evidence of beta-amyloid-induced immune dysfunction in AD and AD models. In addition to the well-studied AD pathology-related alterations in microglial phenotype and function (43, 44), we provide new insights into beta-amyloid-induced alterations in APCs and T-cells, key players of brain immune surveillance.

Indeed, genome-wide association studies (GWAS) have shown a link between AD risk and gene variants related to immune response in general and antigen presentation in particular. HLA loci have been consistently identified in GWAS of AD risk, both in late (45) and early onset (46) AD types. A systematic analysis of GWAS for AD pathology-associated pathways highlighted “MHC class II receptor activity” and “antigen processing and presentation” among the most significantly over-represented GO terms (47).

The recent re-discovery of dural lymphatics using transgenic reporter mice and advanced *in vivo* imaging (12, 13, 48, 49) has shed novel light on immune pathways in neurodegeneration. The dural lymphatic system is thought to be among the major CSF efflux pathways toward cervical lymph nodes, together with formerly described routes along the olfactory nerve through the cribriform plate and other cranial nerves (50). The contribution of the different efflux routes is still a matter of debate. But indeed, brain-derived solutes such as tau and Aβ have been shown to drain to deep cervical lymph nodes supposedly via the glymphatic pathway and the above-mentioned CSF draining routes (51, 52). In the deep cervical lymph nodes they can be processed and presented by DCs in order to prime naïve T-cells. In a possible scenario, Aβ oligomers could drain to deep cervical lymph nodes and induce activation of matching T-cells with reactivity toward beta-amyloid-derived epitopes. Those T-cells may reach the CSF via the choroid plexus or by crossing the blood-brain-barrier (BBB), are reactivated by local APCs at the brain boundaries, are licensed to enter the parenchyma and promote phagocytosis of beta-amyloid-derived material via IFNγ release. Indeed, Aβ-specific pro-inflammatory T-cells injected into the cerebral ventricles have been shown to reduce beta-amyloid plaque burden in other APP and PSEN1 transgenic animal models, including the APPPS1-21 (53) and the 5xFAD (26) model. Specifically in the latter model, a mechanism involving upregulation of MHC-II on microglia has been described (26). Impaired brain APCs' function might block the reactivation of surveilling T-cells, thus leading to uncontrolled Aβ deposition.

Here, we asked whether Aβ could directly affect phenotype and function of APCs. To answer this question, we used both *ex vivo* and *in vitro* approaches. *Ex vivo*, we took advantage of the reproducible and time-dependent accumulation of human Aβ in an APP-transgenic animal model to explore how beta-amyloid pathology would influence immune cell phenotypes and numbers. While the APP-PS1 model, like all APP-transgenic mouse models, was not originally designed to address immunological questions, it has been widely used in the field to study the effect of beta-amyloid pathology on a variety of cell types, including microglia (54).

In this model, we observed that the rise of oligomeric, amyloidogenic Aβ1-42 species coincided with a reduced surface MHC-II expression per cell in brain-derived APCs (Figure 2D). In addition, the levels of surface MHC-II and Aβ oligomers were inversely correlated, suggesting a direct link (Figure 2E). Lower per-cell surface expression of MHC-II is typical of immature DCs and indicative of low functionality (42). Nevertheless, previous reports have stated an overall increase of MHC-II protein in hippocampus of patients with mild to moderate AD compared to brains of non-demented controls (55). Those results are consistent with our observation of increasing total numbers of MHC-II+ cells in the brain during beta-amyloid pathology progression (Figure 2C). In fact, microglia have been shown to migrate toward Aβ plaques and surround them within 1–2 days of their formation (4, 8). Moreover, both parenchymal microglia and CNS border-associated macrophages expressing MHC-II have been found clustering around parenchymal and vascular beta-amyloid depositions, respectively (56). However, it remains speculative if the cerebral immune system recruits additional MHC-II+ APCs to beta-amyloid-burdened sites, in order to compensate for the reduced antigen presentation capacity of local APCs.

While APC alterations appeared as early as at 8 months of age, alterations in T-cells were not apparent until 14 months of age (Figure 3). The reduced frequency of pro-inflammatory T-cells might represent a late effect of dysfunctional brain antigen presentation in aged APP-PS1 transgenic mice. Likewise, it might be possible that both effects are unrelated. The causes of the observed MHC-II and T-cell alterations could be various, among them an accelerated aging phenotype of both immune cell types. Nevertheless, whether the accumulation of different aggregated Aβ species mediates immune senescence in physiological or pathological conditions remains to be elucidated. Moreover, both MHC-II levels and pro-inflammatory T-cell frequencies in late disease stages might be also reduced because of direct toxic effect of Aβ or due to chronic antigenic stress leading to immune overreaction and exhaustion (57). Furthermore, chronically high levels of Aβ have been shown to cause immune tolerance or hypo-responsiveness to Aβ epitopes in APP-transgenic mice (58) and given the early presence of cerebral amyloid angiopathy (CAA) in our model (59), it is possible that Aβ species, including oligomeric forms, might drain constantly into the periphery inducing long-term immune exhaustion.

Our subsequent *in vitro* experiments were geared to clarify these points. By using a well-established assay of antigen presentation (31) we tested the hypothesis that Aβ has a direct and acute effect on antigen-presenting cells. We proved that acute treatment with *in vitro* produced Aβ1-42 oligomers (but not amylin oligomers or the largely monomeric Aβ1-42 scrambled control) can impair antigen presentation by acting on DCs directly. Most probably, this effect is caused by specific conformational changes of Aβ. In fact, *in vitro* produced low-molecular weight (8–70 kDa) oligomeric structures of Aβ from human AD brains have been shown to be most immune-reactive (35). However, our choice of control (scrambled control peptide) did not allow us to establish this specifically. Overall, further *in vitro* studies using different well-characterized Aβ aggregation states and species would be needed to elucidate the exact process. Surprisingly, the effect on APCs in this experimental *in vitro* setting was independent on MHC-II surface levels, as opposed to what we observed in *ex vivo* experiments. We speculate that the acute treatment used in the *in vitro* experiments cannot fully recapitulate the *in vivo* setting, with continuous and prolonged exposure to aggregated Aβ species for many months. It is very likely that the chronic exposure to different Aβ aggregation forms *in vivo* leads to further exhaustion of APCs, which is not reflected in the *in vitro* experiments.

In our search for a mechanism explaining the inhibitory effect of Aβ on DCs, we performed RNAseq analysis of oligomeric Aβ-treated cells. We provide here the first characterization of Aβ-induced effects on immune gene expression in DCs. Our results indicate that Aβ is not overly toxic to DCs, but selectively targets the transcription of genes related to a variety of cellular functions. We identified a number of genes in GO terms “innate immune response” and “cellular response to TNF” that were significantly altered by Aβ oligomer treatment. Among these genes, we observed a down-regulation of important chemokines, such as CCL8, a member of the monocyte chemoattractant protein (MCP) family, also known as MCP-2. Murine CCL8 acts as a CCR1, CCR2b, CCR5, and CCR8 agonist (60, 61), attracting regulatory T-cells, CD4+ thymocytes, monocyte-derived DCs and macrophages. Further downregulated chemokines upon Aβ treatment were CCL2 (MCP-1) and CCL7. The role of CCL2, the agonist of CCR2 receptors on monocytes and macrophages, is a matter of debate in AD research. Some studies have found elevated protein levels of CCL2 in human AD brain tissue (62, 63) suggesting it as a marker for AD severity. However, other studies have shown that the CCL2/CCR2 axis might be impaired in AD pathology, thus preventing chemotaxis of monocyte-derived phagocytes to sites of beta-amyloid accumulation (64). Importantly, another down-regulated gene was *Tnfsf18* (Tumor Necrosis Factor (Ligand) Superfamily, Member 18), expressing a cytokine that binds to glucocorticoid-induced TNFR-related receptor (GITR) and regulates multiple T-cell responses. As co-stimulator it was shown to lower the threshold for T-cell activation and T-cell proliferation (65). It is also involved in cell-adhesion by up-regulating the expression of VCAM-1 and ICAM-1 (66), both involved in the formation of the immunological synapse between T-cells and APCs (67). Unsurprisingly, the GO term “amyloid-beta binding” was also amongst the pathways affected by Aβ. It is interesting to note that an additional GO term profoundly impacted by Aβ was “extracellular exosome.” DC-derived exosomes have been shown to carry antigens and functional MHC peptide complexes (68). It remains the possibility that Aβ inhibits BM-DCs' antigen presentation via changes in exosome composition and release. Indeed, “plasma membrane” and “external side of plasma membrane” were amongst the top 10 GO terms affected by Aβ. It is also interesting to note that “myelin sheath” appeared as one of the top 10 GO terms for cellular compartment. While overall toxicity of Aβ to oligodendrocytes has been described (69), Aβ-induced alterations in myelin sheath-related genes in oligodendrocytes remains to be elucidated.

In sum, we show here for the first time that Aβ affects gene expression in primary DCs, with a number of pathways potentially responsible for reduced antigen presentation activity. Further experiments will be required to establish the exact contribution of each pathway to impaired antigen presentation.

In summary, we propose dysfunctional antigen presentation and reduced T-cell activation as novel downstream elements of beta-amyloid pathology. The concept of Aβ-mediated reduced immune surveillance might have important implications. In cancer, impaired antigen presentation, with consequent dysfunction of CNS-associated T-cell functions, leads to immune evasion and uncontrolled growth of neoplastic cells (70). Similarly, in AD, impaired antigen presentation might cause immune evasion of soluble amyloidogenic oligomers, leading to beta-amyloid build-up and its downstream toxicity. As overt T-cell reaction to Aβ and antibody-dependent cellular cytotoxicity (ADCC) in vessels have been linked to meningoencephalitis (11, 71), novel therapeutic approaches should aim at moderately and selectively re-activate the brain's immune system. A detailed characterization of AD-associated immune populations across the AD continuum holds great promise for the identification of novel biomarkers of disease progression, as well as pharmacological targets.

## Data Availability Statement

The raw RNA sequencing data that support the findings of this study are publicly available (NCBI-GEO, accession number *GSE136789*). All of the code that was used for extended data analysis is available online as R or Python packages (see Supplementary Materials and Methods). All other original datasets are available from the corresponding author upon reasonable request.

## Ethics Statement

The animal study was reviewed and approved by Swiss cantonal veterinary office (Canton Zurich, license numbers 145/2014 and 064/2017).

## Author Contributions

RN and MF conceived and jointly directed the study. CG and MF designed the experimental setup. CG and AM performed the experiments, analyzed the data and co-wrote the manuscript. BE and MF provided conceptual assistance and substantively revised the manuscript. All authors read and approved the manuscript.

## Conflict of Interest

RN is member of the board of directors of Neurimmune AG. MF is the co-founder and CSO of the Women's Brain Project. She received consulting fees from Eli Lilly & Co. for a project unrelated to the present paper. The remaining authors declare that the research was conducted in the absence of any commercial or financial relationships that could be construed as a potential conflict of interest.
