# Supplementary material for: Oligomeric Forms of Human Amyloid-Beta(1–42) Inhibit Antigen Presentation
Source: Front Immunol. 2020 Jun 5;11:1029. doi: 10.3389/fimmu.2020.01029 (PMC7290131; doi:10.3389/fimmu.2020.01029)
Supplement: Supplementary file 1 [file Data_Sheet_1.pdf]

Supplementary Material

1 Supplementary Materials and Methods

Detailed quality control and data analysis for RNA sequencing. The quality of RNAseq data was assessed using fastqc (v. 0.11.5, http://www.bioinformatics.babraham.ac.uk/projects/fastqc/) and RSeQC (Python package version 2.6.4, http://rseqc.sourceforge.net/). The reads were mapped to the reference genome using HiSat2 (R package version 1.0.3, DOI: 10.18129/B9.bioc.Rhisat2). FeatureCounts (version 1.6.0, part of the 'Rsubread' R package, DOI: 10.18129/B9.bioc.Rsubread) was used to count the number of reads overlapping with each gene as specified in the genome annotation (Mus_musculus.GRCm38.91). The R package DESeq2 (version 1.24.0, DOI: 10.18129/B9.bioc.DESeq2) was used to test for differential gene expression between the experimental groups. The log-fold change of each gene was adjusted to include the evidence based on which the log-fold change was estimated and to extrapolate the adjusted p-value (adjp). A false discovery rate correction was applied using DESeq2, based on the Benjamini-Hochberg procedure. The gene ontology (GO) enrichment analysis was performed with topGO (R package version 2.24.0, DOI: 10.18129/B9.bioc.topGO) and GOSeq (R package version 1.36.0, DOI: 10.18129/B9.bioc.goseq). These tools were used to identify gene ontology terms and KEGG (Kyoto Encyclopedia of Genes and Genomes) pathways containing unusual differentially expressed genes. Genes were ranked within a GO term using a two-fold approach including (1) Fisher's exact test, comparing the proportion of differentially expressed genes among all genes assigned to the GO term with all other genes, and (2) Kolmogorov-Smirnov test, which orders all genes by p-value and tests if the genes assigned to a particular GO term are enriched at the top or the bottom of this ranking. All analyses were run in R (version 3.4.4). The top 10 GO terms of each category (Biological Process, Cellular Compartment and Molecular Function) for each BM-DC treatment comparison were listed

based on the assigned p-value. The overall 30 top GO hits (top10 GO terms for Biological Process + top10 GO terms for Cellular Compartment + top10 GO terms for Molecular Function) from each comparison were grouped using a Venn diagram to identify GO terms specifically enriched in each individual BM-DC treatment comparison. RNAseq data were mapped in volcano plots. In detail, each gene was plotted according to the log_2(fold change) and to the negative log_{10}(adjp). A significance threshold was set at $adjp < 0.05$. Volcano plots were generated using GraphPad Prism (Version 8.21, GraphPad Software).

2 Supplementary Figures and Tables

2.1 Supplementary Figures

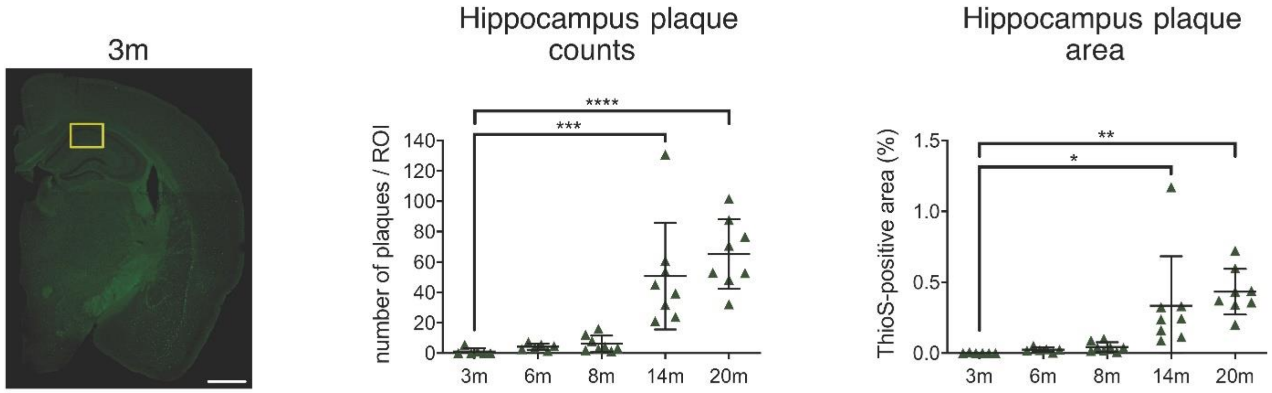

Figure S1. Increase of hippocampal beta-amyloid plaque load during pathology progression in APP-PS1 transgenic mice. Thioflavin-S staining of 40µm coronal sections. Scale bar = 0.8mm. Yellow box (0.33mm^2) shows area of interest for quantification within CA1 region of mouse anterior hippocampus. Quantitative analysis of plaque load shows increasing numbers and covering area of beta-amyloid plaques in the hippocampus starting at 14 months of age. Each symbol represents data from one tg mouse. 3m, n=8; 6m, n=8; 8m, n=8; 14m, n=8; 20m, n=8.

(Data are shown as mean ± SD, *=p<0.05, **=p<0.01, ***=p<0.001, ****=p<0.0001, one-way ANOVA).

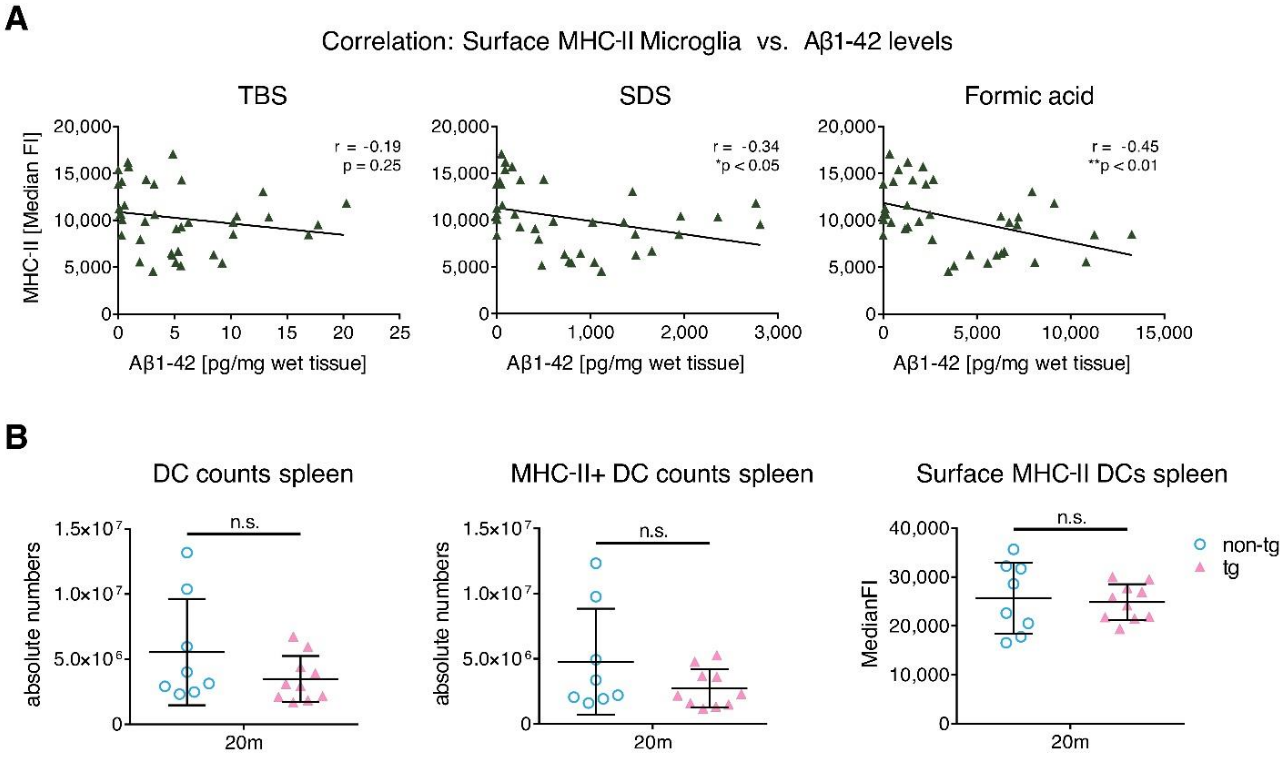

Figure S2. Inverse correlation between microglial MHC-II surface expression and aggregated Aβ1-42 levels; no effect of brain beta-amyloidosis on splenic DCs. (A) Increased levels of SDS- and formic acid-soluble amyloidogenic Aβ1-42 species, but not TBS-soluble forms, significantly correlate with diminished MHC-II surface expression on microglia from brains of APP-PS1 tg mice. Each symbol represents data from one tg mouse (n=37). (Pearson's correlation, Pearson coefficient r indicates negative correlation, two-tailed p-value testing).

(B) Late-stage beta-amyloid pathology in brains of APP-PS1 tg mice has no impact on absolute DC numbers, numbers of MHC-II+ DCs or surface MHC-II expression per DC (measured by median fluorescence intensity = MedianFI) in the spleen compared to non-tg littermates. Each symbol represents data from one mouse. 20m, n=8 (non-tg), n=10 (tg).

(Data are shown as mean ± SD, n.s. = non-significant, two-way ANOVA with Bonferroni's multiple comparisons test).

A

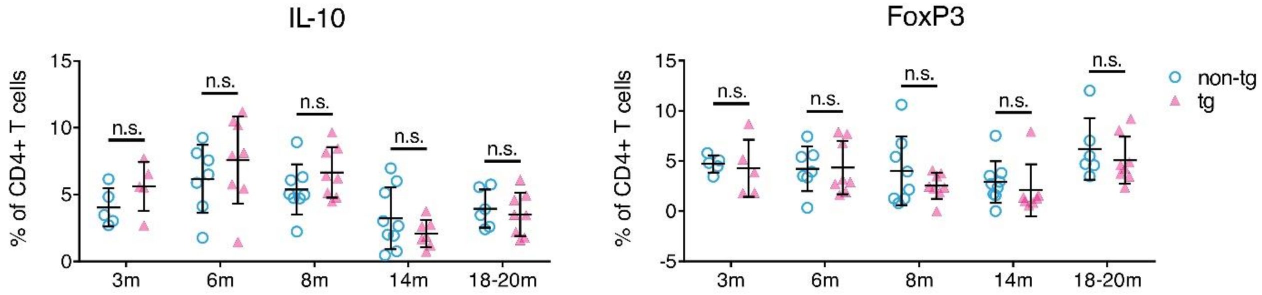

IL-10
FoxP3
% of CD4+ T cells
n.s.
3m
6m
8m
14m
18-20m
non-tg
tg

B

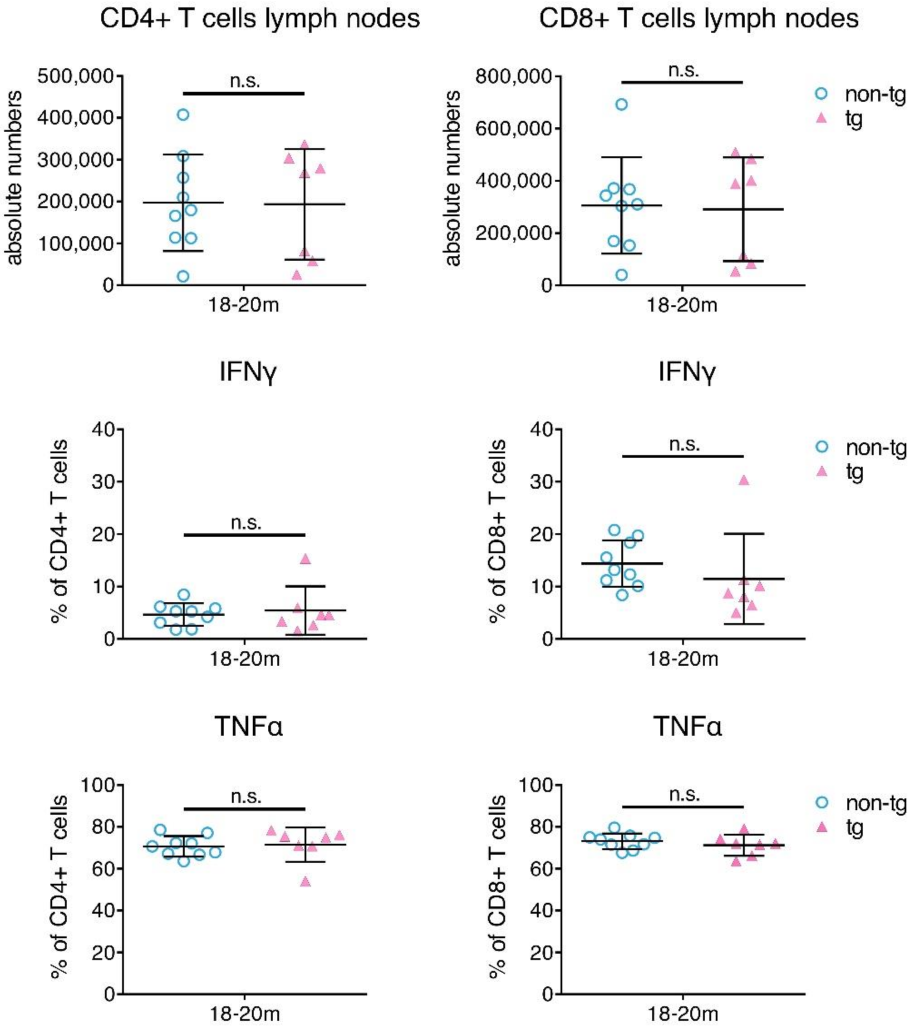

CD4+ T cells lymph nodes
CD8+ T cells lymph nodes
absolute numbers
IFNγ
TNFα
% of CD4+ T cells
% of CD8+ T cells
n.s.
18-20m
non-tg
tg

Figure S3. Unaffected tolerogenic CD4+ T-cell responses in brains of APP-PS1 transgenic mice; no effect of cerebral beta-amyloidosis on T-cells from inguinal lymph nodes. (A) Flow cytometry analysis of intracellular IL-10 and FoxP3-content reveals that cerebral beta-amyloidosis has no effect on tolerogenic (Th2 and regulatory) CD4+ T-cell responses in brains of APP-PS1 tg animals compared to non-tg animals. Each symbol represents data from one mouse. 3m, n=5 (non-tg), n=5 (tg); 6m, n=7 (non-tg), n=9 (tg); 8m, n=8 (non-tg), n=7 (tg); 14m, n=9 (non-tg), n=7 (tg); 18-20m, n=6 (non-tg), n=8 (tg).

(Data are shown as mean ± SD, n.s. = non-significant, two-way ANOVA with Bonferroni's multiple comparisons test).

(B) Late-stage beta-amyloid pathology in APP-PS1 tg mice has no significant impact on absolute CD4+ and CD8+ T-cell numbers and frequency of IFNγ- or TNFα-secreting T-cells compared to non-tg littermates. Each symbol represents data from one mouse. 18-20m, n=9 (non-tg), n=7 (tg).

(Data are shown as mean ± SD, n.s. = non-significant, two-way ANOVA with Bonferroni's multiple comparisons test).

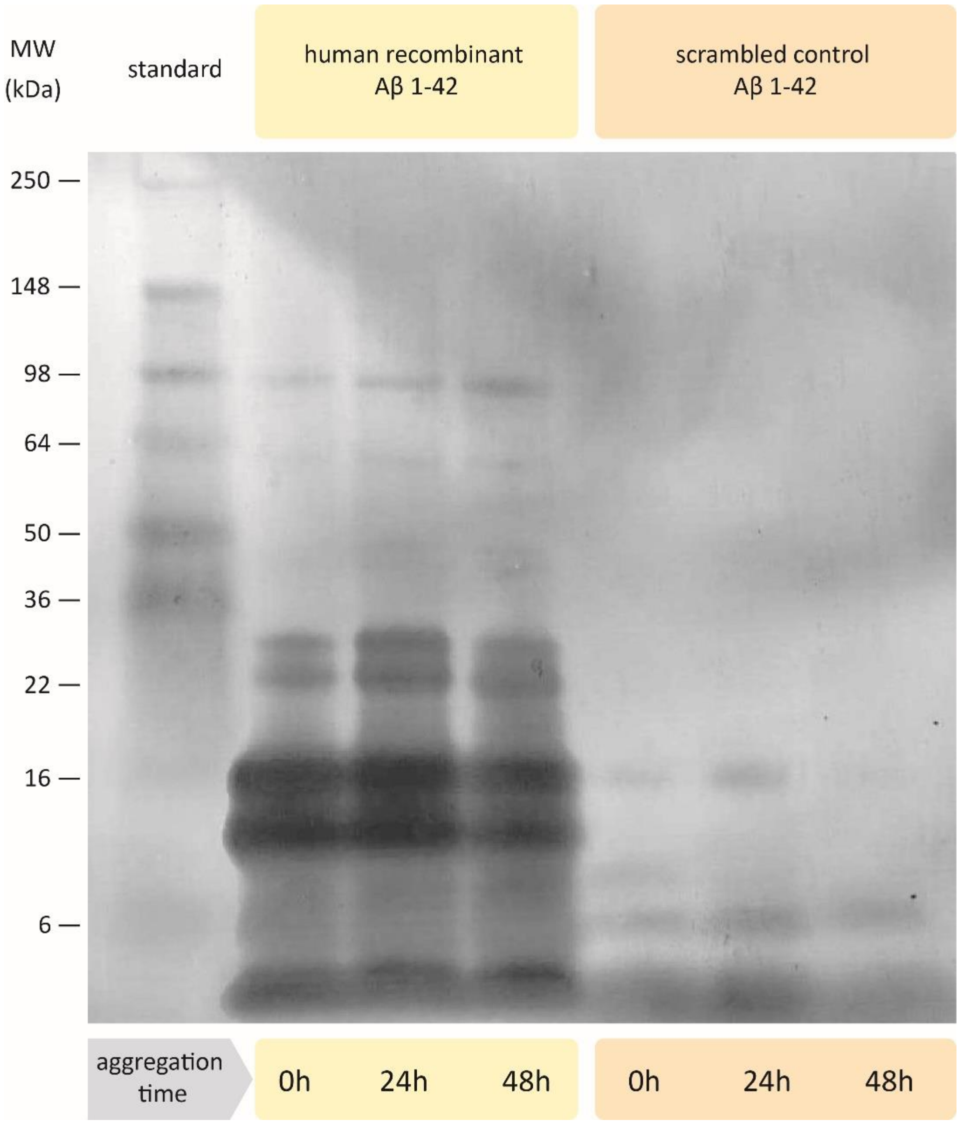

Figure S4. Examination of the aggregation state of Aβ1-42 preparations. After applying the oligomeric Aβ1-42 preparation protocol with different aggregation times (0, 24 and 48 hours), human recombinant Aβ1-42 (20 μg per lane) and scrambled control (9 μg per lane) have been loaded on a 10-20% tris-glycine gel, followed by silver staining. Monomeric residues at approximately 4.5kDa (MW of Aβ1-42) as well as low-molecular weight oligomers in form of trimers at 13.5kDa and tetramers at 18kDa are the most evident Aβ1-42 species. As described in previous studies, scrambled control peptides show minor aggregation capability.

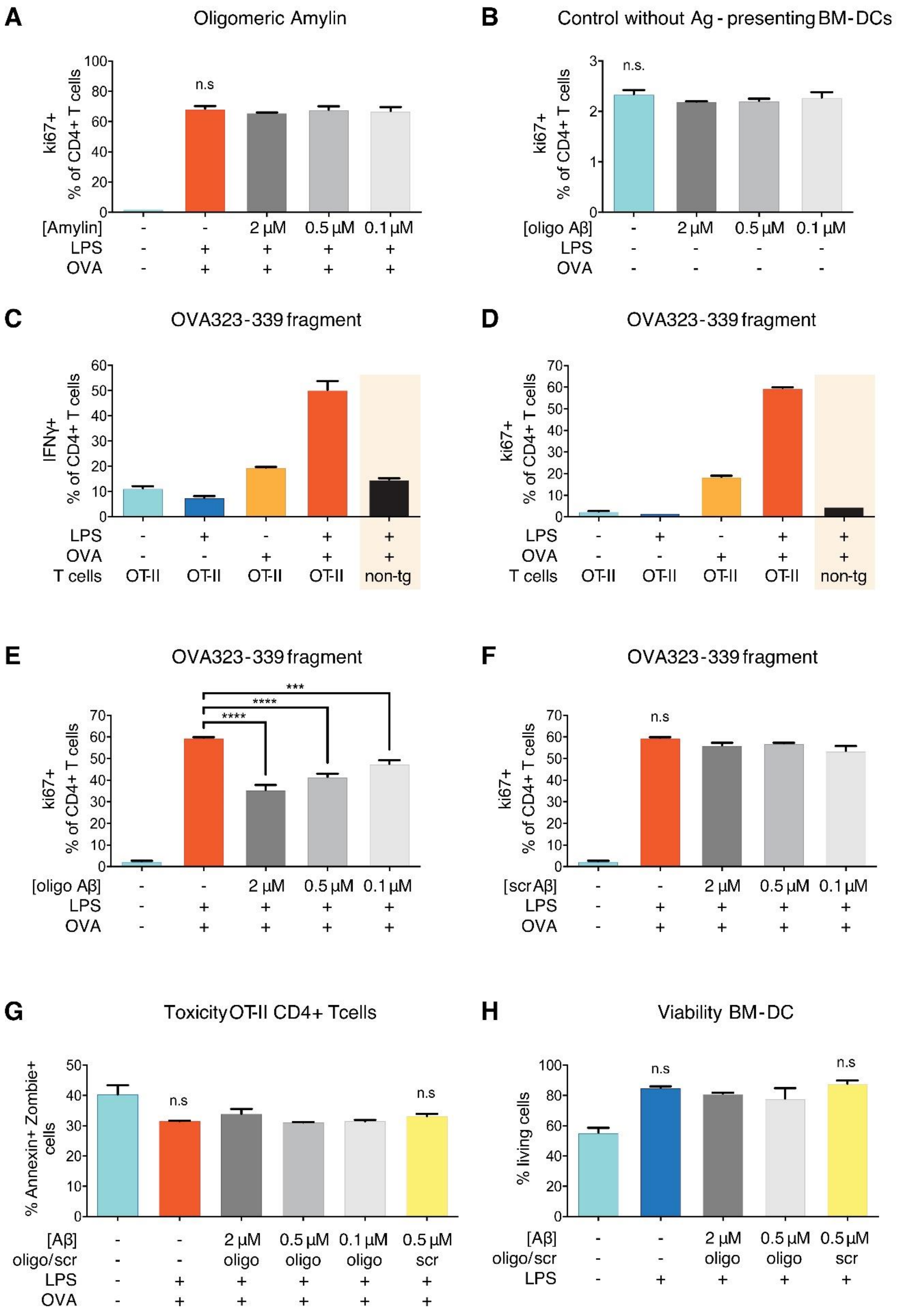
A
Oligomeric Amylin
ki67+
% of CD4+ T cells
100
80
60
40
20
0
n.s
[Amylin] - - 2 µM 0.5 µM 0.1 µM
LPS - + + + +
OVA - + + + +
B
Control without Ag - presenting BM-DCs
ki67+
% of CD4+ T cells
3
2
1
0
n.s.
[oligo Aβ] - 2 µM 0.5 µM 0.1 µM
LPS - - - -
OVA - - - -
C
OVA323-339 fragment
IFNγ+
% of CD4+ T cells
60
50
40
30
20
10
0
LPS - + - + +
OVA - - + + +
T cells OT-II OT-II OT-II OT-II non-tg
D
OVA323-339 fragment
ki67+
% of CD4+ T cells
70
60
50
40
30
20
10
0
LPS - + - + +
OVA - - + + +
T cells OT-II OT-II OT-II OT-II non-tg
E
OVA323-339 fragment
ki67+
% of CD4+ T cells
70
60
50
40
30
20
10
0
***
****
****
[oligo Aβ] - - 2 µM 0.5 µM 0.1 µM
LPS - + + + +
OVA - + + + +
F
OVA323-339 fragment
ki67+
% of CD4+ T cells
70
60
50
40
30
20
10
0
n.s
[scr Aβ] - - 2 µM 0.5 µM 0.1 µM
LPS - + + + +
OVA - + + + +
G
Toxicity OT-II CD4+ Tcells
% Annexin+ Zombie+ cells
50
40
30
20
10
0
n.s
n.s
[Aβ] - - 2 µM 0.5 µM 0.1 µM 0.5 µM
oligo/scr - - oligo oligo oligo scr
LPS - + + + + +
OVA - + + + + +
H
Viability BM-DC
% living cells
100
80
60
40
20
0
n.s
n.s
[Aβ] - - 2 µM 0.5 µM 0.5 µM
oligo/scr - - oligo oligo scr
LPS - + + + +

Figure S5. Aβ1-42 oligomer-specific inhibition of antigen-presentation *in vitro* without involvement of antigen-processing machinery or cell toxicity. LPS-matured bone marrow-derived dendritic cells (BM-DCs) present OVA-derived antigens to OVA-specific OT-II tg CD4+ T-cells. **(A)** Human aggregated Amylin1-37 has no effect on frequency of ki67+ OT-II CD4+ T-cells. **(B)** Incubation of OT-II CD4+ T-cells with Aβ1-42 oligomers but without antigen-presenting BM-DCs does not affect T-cell proliferation in terms of ki67 expression. **(C, D)** Substitution of full-length OVA antigen with OVA 323-339 fragment, the antigenic CD4+ T-cell epitope, leads to a similar OT-II T-cell activation response as seen for full-length OVA. **(E)** Addition of Aβ1-42 oligomers, but not scrambled (scr) peptide **(F)**, reduces frequency of ki67+ OT-II CD4+ T-cells with dose-response effect. **(G)** Apoptosis-marker AnnexinV and combined dead cell staining ('Zombie') show that reduced T-cell activation is not a result of Aβ1-42 toxicity towards OT-II CD4+ T-cells. **(H)** Analysis of BM-DC viability shows no effect of Aβ1-42 oligomer treatment compared to scr peptide or untreated cells during the course of antigen-presentation assay. Representative results of at least 3 independent experiments are shown in each graph; $n \geq 2$ per treatment condition in each experiment.

(Data are shown as mean ± SD, ***=$p<0.001$, ****=$p<0.0001$, n.s. = non-significant, one-way ANOVA).

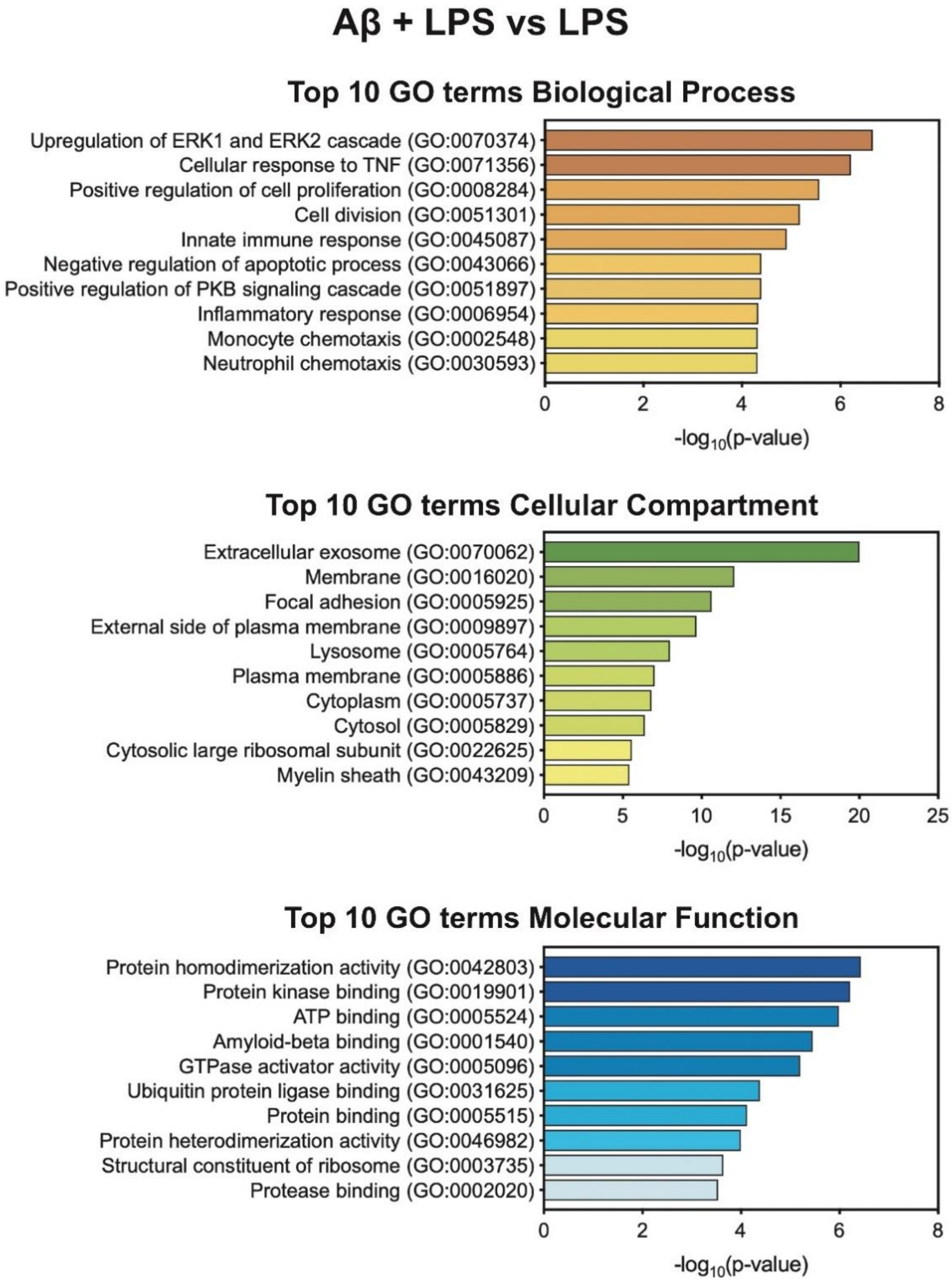

Figure S6. Top 10 GO terms affected by Aβ+LPS treatment compared to LPS-matured cells. The top 10 GO terms identified in the 'Aβ+LPS vs LPS' comparison within the three GO categories (biological process, cellular compartment and molecular function) are ranked according to their p-values.

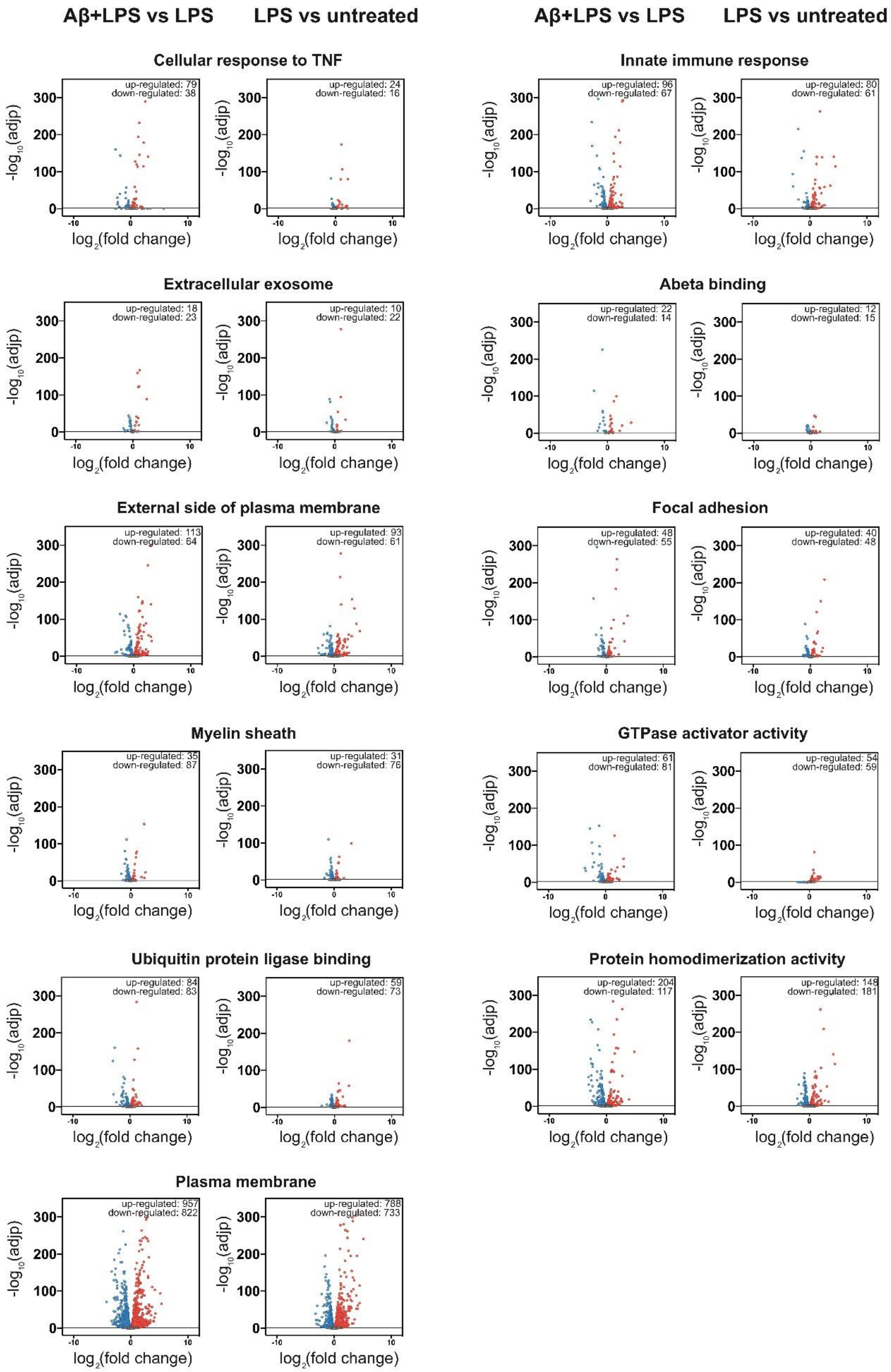
Aβ+LPS vs LPS
LPS vs untreated
Aβ+LPS vs LPS
LPS vs untreated
Cellular response to TNF
up-regulated: 79
down-regulated: 38
up-regulated: 24
down-regulated: 16
Innate immune response
up-regulated: 96
down-regulated: 67
up-regulated: 80
down-regulated: 61
Extracellular exosome
up-regulated: 18
down-regulated: 23
up-regulated: 10
down-regulated: 22
Abeta binding
up-regulated: 22
down-regulated: 14
up-regulated: 12
down-regulated: 15
External side of plasma membrane
up-regulated: 113
down-regulated: 64
up-regulated: 93
down-regulated: 61
Focal adhesion
up-regulated: 48
down-regulated: 55
up-regulated: 40
down-regulated: 48
Myelin sheath
up-regulated: 35
down-regulated: 87
up-regulated: 31
down-regulated: 76
GTPase activator activity
up-regulated: 61
down-regulated: 81
up-regulated: 54
down-regulated: 59
Ubiquitin protein ligase binding
up-regulated: 84
down-regulated: 83
up-regulated: 59
down-regulated: 73
Protein homodimerization activity
up-regulated: 204
down-regulated: 117
up-regulated: 148
down-regulated: 181
Plasma membrane
up-regulated: 957
down-regulated: 822
up-regulated: 788
down-regulated: 733
-log10(adjp)
log2(fold change)

Figure S7. Gene expression changes of GO terms specifically affected in the ‘Aβ+LPS vs LPS’ comparison. Volcano plots summarizing the expression level changes of genes belonging to 11 GO terms specifically enriched in ‘Aβ+LPS vs LPS’. Gene expression changes for ‘Aβ+LPS vs LPS’ are depicted alongside ‘LPS vs Untreated’ in order to compare the impact of Aβ treatment to LPS-maturation only. Blue = down-regulated, red = up-regulated. Significance is considered at adjp < 0.05.

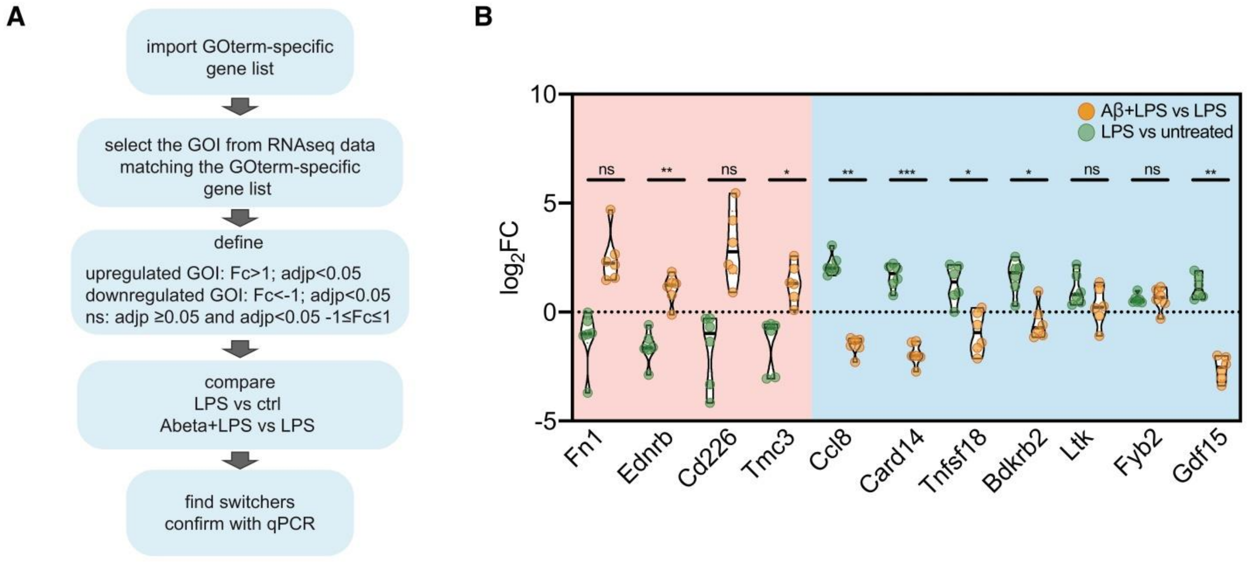

Figure S8. GO analysis strategy and qPCR verification for genes of interest. (A) Consecutive strategy for GO term analysis. GOI = gene of interest, Fc = fold change, adjp = adjusted p value, ns = non-significant. **(B)** Verification of Aβ-induced changes in GOI via qPCR. 11 GOI belonging to GO terms 'innate immune response', 'cellular response to TNF' and 'plasma membrane', which are strongly affected by Aβ treatment according to RNAseq data, are separately analyzed by qPCR. Fold changes are depicted for 'LPS vs Untreated' and 'Aβ+LPS vs LPS' comparisons. GOI, which according to RNAseq data originally switched from down- to up-regulation, are depicted in the red area, GOI that originally switched from up- to down-regulation are shown in the blue area. Each comparison includes 6 data points: 3 samples per condition x qPCR in duplicates.

(Data are shown as violin plots with indicated median line; minimum and maximum extension represent lowest and highest value; dotted lines represent quartiles; *=p<0.05, **=p<0.01, ***=p<0.001, n.s. = non-significant; multiple t-tests with Holm-Sidak post-hoc test).

2.2 Supplementary Tables

Table S1. Complete list of adjusted p-values for cortex plaque counts (shown in Figure 1B). Statistical analysis of cortex plaque counts in APP-PS1 transgenic mice at 3, 6, 8, 14 and 20 months of age via one-way ANOVA and Bonferroni's multiple comparisons correction. ***=p<0.001, ****=p<0.0001.

**Cortex plaque counts**	**3m**	**6m**	**8m**	**14m**	**20m**
**3m**	-	> 0.9999	> 0.9999	**** < 0.0001	**** < 0.0001
**6m**	> 0.9999	-	> 0.9999	**** < 0.0001	**** < 0.0001
**8m**	> 0.9999	> 0.9999	-	*** 0.0003	**** < 0.0001
**14m**	**** < 0.0001	**** < 0.0001	*** 0.0003	-	0.1418
**20m**	**** < 0.0001	**** < 0.0001	**** < 0.0001	0.1418	-

Table S2. Complete list of adjusted p-values for cortex plaque area (shown in Figure 1B). Statistical analysis of cortex plaque area (in %) in APP-PS1 transgenic mice at 3, 6, 8, 14 and 20 months of age via one-way ANOVA and Bonferroni's multiple comparisons correction. *=p<0.05, **=p<0.01, ***=p<0.001.

**Cortex plaque area**	**3m**	**6m**	**8m**	**14m**	**20m**
**3m**	-	> 0.9999	> 0.9999	* 0.0150	*** 0.0002
**6m**	> 0.9999	-	> 0.9999	* 0.0411	*** 0.0006
**8m**	> 0.9999	> 0.9999	-	0.1088	** 0.0013
**14m**	* 0.0150	* 0.0411	0.1088	-	> 0.9999
**20m**	*** 0.0002	*** 0.0006	** 0.0013	> 0.9999	-

Table S3. Complete list of adjusted p-values for hippocampus plaque counts (shown in Figure S1). Statistical analysis of hippocampus plaque counts in APP-PS1 transgenic mice at 3, 6, 8, 14 and 20 months of age via one-way ANOVA and Bonferroni's multiple comparisons correction. **=p<0.01, ***=p<0.001, ****=p<0.0001.

**Hippo-campus plaque counts**	**3m**	**6m**	**8m**	**14m**	**20m**
**3m**	-	> 0.9999	> 0.9999	*** 0.0007	**** < 0.0001
**6m**	> 0.9999	-	> 0.9999	** 0.0017	**** < 0.0001
**8m**	> 0.9999	> 0.9999	-	** 0.0011	**** < 0.0001
**14m**	*** 0.0007	** 0.0017	** 0.0011	-	> 0.9999
**20m**	**** < 0.0001	**** < 0.0001	**** < 0.0001	> 0.9999	-

Table S4. Complete list of adjusted p-values for hippocampus plaque area (shown in Figure S1). Statistical analysis of hippocampus plaque area (in %) in APP-PS1 transgenic mice at 3, 6, 8, 14 and 20 months of age via one-way ANOVA and Bonferroni's multiple comparisons correction. *=p<0.05, **=p<0.01.

**Hippo-campus plaque area**	**3m**	**6m**	**8m**	**14m**	**20m**
**3m**	-	> 0.9999	> 0.9999	* 0.0213	** 0.0014
**6m**	> 0.9999	-	> 0.9999	* 0.0395	** 0.0027
**8m**	> 0.9999	> 0.9999	-	* 0.0351	** 0.0019
**14m**	* 0.0213	* 0.0395	* 0.0351	-	> 0.9999
**20m**	** 0.0014	** 0.0027	** 0.0019	> 0.9999	-

Table S5. Complete list of adjusted p-values for MSD-analysis of protein extracts from prefrontal cortex (shown in Figure 1D). Statistical analysis of TBS-soluble Aβ1-38 protein levels from prefrontal cortex of APP-PS1 transgenic mice at 3, 6, 8, 14 and 20 months of age via one-way ANOVA and Bonferroni's multiple comparisons correction. *=p<0.05, ****=p<0.0001.

**Aβ1-38 TBS fraction**	**3m**	**6m**	**8m**	**14m**	**20m**
**3m**	-	> 0.9999	> 0.9999	* 0.0189	**** < 0.0001
**6m**	> 0.9999	-	> 0.9999	* 0.0360	**** < 0.0001
**8m**	> 0.9999	> 0.9999	-	0.2086	**** < 0.0001
**14m**	* 0.0189	* 0.0360	0.2086	-	**** < 0.0001
**20m**	**** < 0.0001	**** < 0.0001	**** < 0.0001	**** < 0.0001	-

Table S6. Complete list of adjusted p-values for MSD-analysis of protein extracts from prefrontal cortex (shown in Figure 1D). Statistical analysis of SDS-soluble Aβ1-38 protein levels from prefrontal cortex of APP-PS1 transgenic mice at 3, 6, 8, 14 and 20 months of age via one-way ANOVA and Bonferroni's multiple comparisons correction. **=p<0.01, ****=p<0.0001.

**Aβ1-38 SDS fraction**	**3m**	**6m**	**8m**	**14m**	**20m**
**3m**	-	> 0.9999	0.4805	**** < 0.0001	**** < 0.0001
**6m**	> 0.9999	-	0.6882	**** < 0.0001	**** < 0.0001
**8m**	0.4805	0.6882	-	** 0.0069	**** < 0.0001
**14m**	**** < 0.0001	**** < 0.0001	** 0.0069	-	**** < 0.0001
**20m**	**** < 0.0001	**** < 0.0001	**** < 0.0001	**** < 0.0001	-

Table S7. Complete list of adjusted p-values for MSD-analysis of protein extracts from prefrontal cortex (shown in Figure 1D). Statistical analysis of formic acid-soluble Aβ1-38 protein levels from prefrontal cortex of APP-PS1 transgenic mice at 3, 6, 8, 14 and 20 months of age via one-way ANOVA and Bonferroni's multiple comparisons correction. **=p<0.01, ***=p<0.001, ****=p<0.0001.

**Aβ1-38 formic acid fraction**	**3m**	**6m**	**8m**	**14m**	**20m**
**3m**	-	> 0.9999	> 0.9999	****<br>< 0.0001	****<br>< 0.0001
**6m**	> 0.9999	-	> 0.9999	***<br>0.0001	****<br>< 0.0001
**8m**	> 0.9999	> 0.9999	-	**<br>0.0013	****<br>< 0.0001
**14m**	****<br>< 0.0001	***<br>0.0001	**<br>0.0013	-	***<br>0.0005
**20m**	****<br>< 0.0001	****<br>< 0.0001	****<br>< 0.0001	***<br>0.0005	-

Table S8. Complete list of adjusted p-values for MSD-analysis of protein extracts from prefrontal cortex (shown in Figure 1D). Statistical analysis of TBS-soluble Aβ1-40 protein levels from prefrontal cortex of APP-PS1 transgenic mice at 3, 6, 8, 14 and 20 months of age via one-way ANOVA and Bonferroni's multiple comparisons correction. ****=p<0.0001.

**Aβ1-40 TBS fraction**	**3m**	**6m**	**8m**	**14m**	**20m**
**3m**	-	> 0.9999	> 0.9999	0.0643	**** < 0.0001
**6m**	> 0.9999	-	> 0.9999	0.0941	**** < 0.0001
**8m**	> 0.9999	> 0.9999	-	0.4951	**** < 0.0001
**14m**	0.0643	0.0941	0.4951	-	**** < 0.0001
**20m**	**** < 0.0001	**** < 0.0001	**** < 0.0001	**** < 0.0001	-

Table S9. Complete list of adjusted p-values for MSD-analysis of protein extracts from prefrontal cortex (shown in Figure 1D). Statistical analysis of SDS-soluble Aβ1-40 protein levels from prefrontal cortex of APP-PS1 transgenic mice at 3, 6, 8, 14 and 20 months of age via one-way ANOVA and Bonferroni's multiple comparisons correction. **=p<0.01, ****=p<0.0001.

**Aβ1-40 SDS fraction**	**3m**	**6m**	**8m**	**14m**	**20m**
**3m**	-	> 0.9999	> 0.9999	****<br>< 0.0001	****<br>< 0.0001
**6m**	> 0.9999	-	> 0.9999	****<br>< 0.0001	****<br>< 0.0001
**8m**	> 0.9999	> 0.9999	-	**<br>0.0024	****<br>< 0.0001
**14m**	****<br>< 0.0001	****<br>< 0.0001	**<br>0.0024	-	****<br>< 0.0001
**20m**	****<br>< 0.0001	****<br>< 0.0001	****<br>< 0.0001	****<br>< 0.0001	-

Table S10. Complete list of adjusted p-values for MSD-analysis of protein extracts from prefrontal cortex (shown in Figure 1D). Statistical analysis of formic acid-soluble Aβ1-40 protein levels from prefrontal cortex of APP-PS1 transgenic mice at 3, 6, 8, 14 and 20 months of age via one-way ANOVA and Bonferroni's multiple comparisons correction. ***=p<0.001, ****=p<0.0001.

**Aβ1-40 formic acid fraction**	**3m**	**6m**	**8m**	**14m**	**20m**
**3m**	-	> 0.9999	> 0.9999	**** < 0.0001	**** < 0.0001
**6m**	> 0.9999	-	> 0.9999	**** < 0.0001	**** < 0.0001
**8m**	> 0.9999	> 0.9999	-	**** < 0.0001	**** < 0.0001
**14m**	**** < 0.0001	**** < 0.0001	**** < 0.0001	-	*** 0.0003
**20m**	**** < 0.0001	**** < 0.0001	**** < 0.0001	*** 0.0003	-

Table S11. Complete list of adjusted p-values for MSD-analysis of protein extracts from prefrontal cortex (shown in Figure 1D). Statistical analysis of TBS-soluble Aβ1-42 protein levels from prefrontal cortex of APP-PS1 transgenic mice at 3, 6, 8, 14 and 20 months of age via one-way ANOVA and Bonferroni's multiple comparisons correction. *=p<0.05, ***=p<0.001, ****=p<0.0001.

**Aβ1-42 TBS fraction**	**3m**	**6m**	**8m**	**14m**	**20m**
**3m**	-	> 0.9999	* 0.0273	*** 0.0007	**** < 0.0001
**6m**	> 0.9999	-	0.5404	* 0.0198	**** < 0.0001
**8m**	* 0.0273	0.5404	-	> 0.9999	**** < 0.0001
**14m**	*** 0.0007	* 0.0198	> 0.9999	-	**** < 0.0001
**20m**	**** < 0.0001	**** < 0.0001	**** < 0.0001	**** < 0.0001	-

Table S12. Complete list of adjusted p-values for MSD-analysis of protein extracts from prefrontal cortex (shown in Figure 1D). Statistical analysis of SDS-soluble Aβ1-42 protein levels from prefrontal cortex of APP-PS1 transgenic mice at 3, 6, 8, 14 and 20 months of age via one-way ANOVA and Bonferroni's multiple comparisons correction. *=p<0.05, ***=p<0.001, ****=p<0.0001.

**Aβ1-42 SDS fraction**	**3m**	**6m**	**8m**	**14m**	**20m**
**3m**	-	> 0.9999	* 0.0448	*** 0.0003	**** < 0.0001
**6m**	> 0.9999	-	0.1092	*** 0.0006	**** < 0.0001
**8m**	* 0.0448	0.1092	-	0.7118	**** < 0.0001
**14m**	*** 0.0003	*** 0.0006	0.7118	-	*** 0.0001
**20m**	**** < 0.0001	**** < 0.0001	**** < 0.0001	*** 0.0001	-

Table S13. Complete list of adjusted p-values for MSD-analysis of protein extracts from prefrontal cortex (shown in Figure 1D). Statistical analysis of formic acid-soluble Aβ1-42 protein levels from prefrontal cortex of APP-PS1 transgenic mice at 3, 6, 8, 14 and 20 months of age via one-way ANOVA and Bonferroni's multiple comparisons correction. ****=p<0.0001.

**Aβ1-42 formic acid fraction**	**3m**	**6m**	**8m**	**14m**	**20m**
**3m**	-	> 0.9999	0.3163	**** < 0.0001	**** < 0.0001
**6m**	> 0.9999	-	> 0.9999	**** < 0.0001	**** < 0.0001
**8m**	0.3163	> 0.9999	-	**** < 0.0001	**** < 0.0001
**14m**	**** < 0.0001	**** < 0.0001	**** < 0.0001	-	0.1096
**20m**	**** < 0.0001	**** < 0.0001	**** < 0.0001	0.1096	-

Table S14. Detailed list of specific primers for qPCR. Sequences of forward (FW) and reverse (RV) primers for 11 GOI of interest and 2 housekeeping genes (Gapdh and Rplp2) are listed together with amplicon size.

Gene	FW	RV	Amplicon size
Fn1	ATGTGGACCCCTCCTGATAGT	GCCCAGTGATTTCAGCAAAGG	124
Ednrb	GTGGCTTCTTGGGGGTATGG	TCTTAGTGGGTGGCGTCATTA	102
Cd226	TCGCTCAGAGGCCATTACAG	CCCTGGGCTCTTTAAGTGGAA	203
Tmc3	TCATCCCCTGGGAAATGAGGA	TCGGGAAGGACAACAAAGGC	142
Ccl8	CTGGGCCAGATAAGGCTCC	CATGGGGCACTGGATATTGTT	110
Card14	GAACTGTGCCGCATGGATTC	GTTGGTGAAACGGGAGCTATG	189
Tnfsf18	AACCTCACTGTGTGAATACGAC	AGGAATCACTTGGCCGTAGATTA	86
Bdkrb2	ATGTTCAACGTCACCACACAA	GCTGAGGACAAAGAGGTTCTCC	165
Ltk	TGCTGGAACTATTCTTTGCTCC	GGTGTGGGGGTGACTTTCT	111
Fyb2	CACCATCATCAAACCCCGTCT	CTGAGCCGACTTCAATGTCTG	78
Gdf15	CTGGCAATGCCTGAACAACG	GGTCGGGACTTGGTTCTGAG	142
Gapdh	TGGATTTGGACGCATTGGTC	TTTGCACTGGTACGTGTTGAT	211
Rplp2	CGCTACGTCGCCTCTTACC	CTTGTTGAGCCGATCATCGTC	120
